# Spectral CT-based Nomogram for Noninvasive Assessment and Prognostic Prediction of MSI/dMMR in Esophagogastric Junction Adenocarcinoma

**DOI:** 10.1148/rycan.250654

**Published:** 2026-09-04

**Authors:** Yinchen Wu, Kaihe Lin, Na Lin, Yu Lin, Xinyao Huang, Mi Wang, Dejun She, Dairong Cao

**Affiliations:** ^1^Department of Radiology, the First Affiliated Hospital, Fujian Medical University, 20 Cha-Zhong Road, Fuzhou, Fujian 350005, PR China; ^2^Department of Radiology, National Regional Medical Center, Binhai Campus of the First Affiliated Hospital, Fujian Medical University, Fuzhou, China; ^3^Department of Radiology, The First Hospital of Putian City, Putian, China; ^4^Department of Radiology, Zhongshan Hospital Affiliated to Xiamen University, Xiamen, China; ^5^Department of Pathology, the First Affiliated Hospital, Fujian Medical University, Fuzhou, China; ^6^Department of Pathology, National Regional Medical Center, Binhai Campus of the First Affiliated Hospital, Fujian Medical University, Fuzhou, China; ^7^Department of Radiology, Fujian Key Laboratory of Precision Medicine for Cancer, the First Affiliated Hospital, Fujian Medical, Fuzhou, China; ^8^Key Laboratory of Radiation Biology of Fujian Higher Education Institutions, the First Affiliated Hospital, Fujian Medical University, Fuzhou, China

**Keywords:** Esophagogastric Junction Adenocarcinoma, Spectral CT, Nomogram, Primary Neoplasms, CT-Spectral, Neoplasms-Primary, Pathology, Tumor Immune Microenvironment, Pre-clinical Models

## Abstract

**Purpose:**

To establish and evaluate a nomogram based on quantitative spectral CT parameters for predicting microsatellite instability (MSI) and deficient mismatch repair (dMMR) status in patients with esophagogastric junction adenocarcinoma (EGJA).

**Materials and Methods:**

In this study including retrospective and prospective datasets (enrollment period: May 2021 to January 2026), patients from two centers were divided into training, validation, external test, prospective test, and neoadjuvant chemotherapy cohorts. A nomogram was constructed integrating clinical characteristics with quantitative spectral CT parameters. Model efficacy in predicting MSI/dMMR and its associations with disease-free survival were evaluated. Primary statistical methods included logistic and Cox regression analyses.

**Results:**

In total, 511 patients (median age, 67 years [IQR, 60–73 years]; 333 male) were included. The nomogram incorporated sex, clinical N stage, CT attenuation on 40-keV virtual monoenergetic images, and normalized iodine density during the venous phase. It achieved areas under the receiver operating characteristic curve of 0.87 (95% CI: 0.81, 0.93), 0.87 (95% CI: 0.78, 0.96), 0.91 (95% CI: 0.83, 0.99), 0.89 (95% CI: 0.80, 0.98), and 0.86 (95% CI: 0.76, 0.96) across the five respective cohorts: training, validation, external test, prospective test, and neoadjuvant chemotherapy. In the external test cohort, the nomogram correctly identified 9.76% (four of 41) of the patients misclassified with preoperative biopsy. Furthermore, it stratified patients into distinct disease-free survival risk groups in the training (hazard ratio, 2.04 [95% CI: 1.35, 3.09]; *P* < .001) and validation (hazard ratio, 2.97 [95% CI: 1.46, 6.05]; *P* = .003) cohorts.

**Conclusion:**

The spectral CT–based nomogram enabled preoperative prediction of MSI/dMMR status and prognostic assessment in patients with EGJA.

**Keywords:** Esophagogastric Junction Adenocarcinoma, Spectral CT, Nomogram, Primary Neoplasms, CT-Spectral, Neoplasms-Primary, Pathology, Tumor Immune Microenvironment, Pre-clinical Models

Chinese Clinical Trial Registry identifier nos. ChiCTR2500097335 and ChiCTR2500101639

*Supplemental material is available for this article.*

© The Author(s) 2026. Published by the Radiological Society of North America under a CC BY 4.0 license.

See also the commentary by Méndez and Nava in this issue.

## Graphical abstract

**Figure:**
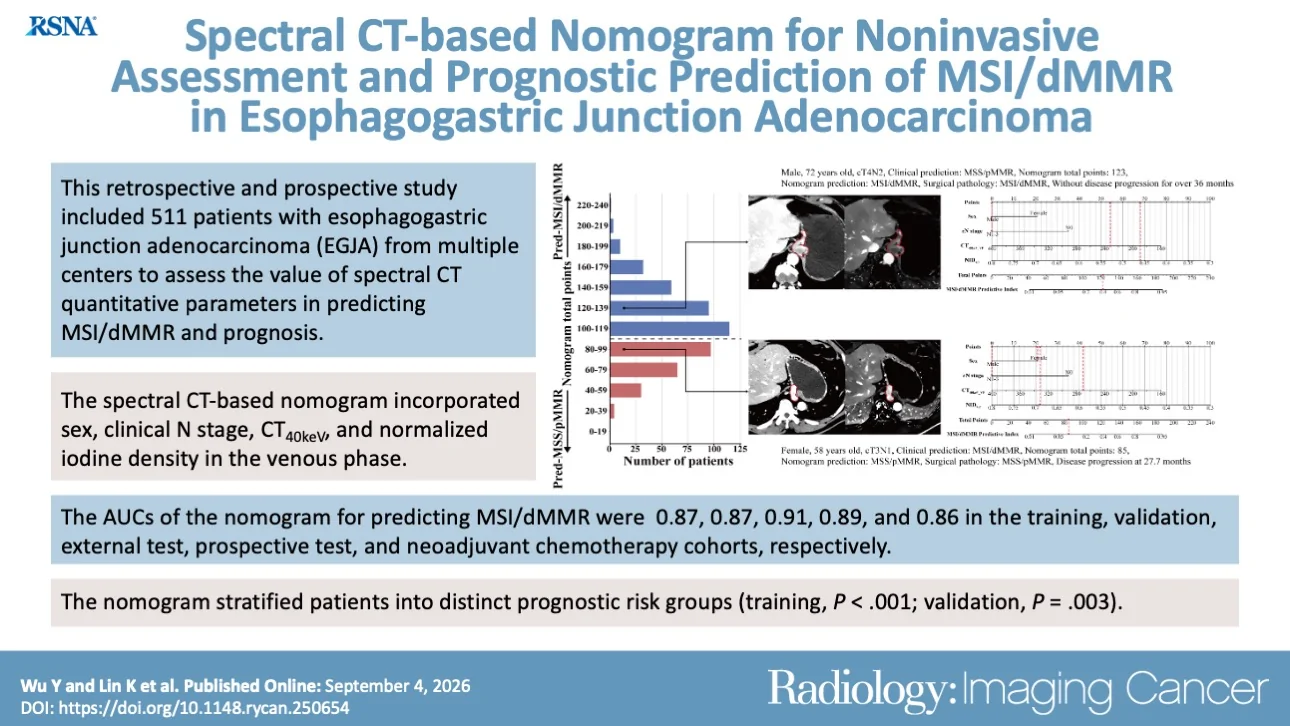

**Summary**

A spectral CT–based nomogram predicted microsatellite instability and deficient mismatch repair status and stratified prognosis in patients with esophagogastric junction adenocarcinoma across retrospective and prospective cohorts.

**Key Points**

■ In this multicenter study of 511 patients with esophagogastric junction adenocarcinoma (EGJA), a spectral CT–based nomogram incorporating sex, clinical N stage, CT attenuation on 40-keV images, and normalized iodine density during the venous phase was developed.
■ The nomogram achieved areas under the receiver operating characteristic curve ranging from 0.86 to 0.91 for predicting microsatellite instability and deficient mismatch repair status across five cohorts.
■ The nomogram stratified patients into distinct prognostic risk groups (training, *P* < .001; validation, *P* = .003).

## Introduction

Esophagogastric junction adenocarcinoma (EGJA) is an independent tumor type with a specific anatomic location and distinct histologic features that differentiate it from esophageal and gastric cancers (1–3). It is a highly deadly cancer (4,5), and up to half of patients with EGJA experience disease recurrence despite receiving standardized, multimodal treatments recommended by clinical guidelines (6,7). The mismatch repair system preserves genomic integrity by correcting DNA replication errors and contributing to other DNA repair pathways (8). Deficient mismatch repair (dMMR), which typically is identified via immunohistochemistry, leads to an accumulation of uncorrected mutations. This causes high mutation rates in short, repetitive DNA sequences, resulting in microsatellite instability (MSI), which is identified by polymerase chain reaction or next-generation sequencing (9). The MSI/dMMR status typically coexists with an intact immune system and fosters a microenvironment characterized by high immune infiltration, thereby driving improved survival and a favorable prognosis in patients with EGJA (10–12). Current diagnostic and treatment guidelines recommend the early detection of MSI/dMMR status to guide clinicians in making differentiated preoperative treatment decisions (1). Immunotherapy is the preferred preoperative treatment for patients with MSI/dMMR who derive no benefit from conventional chemotherapy-based regimens and may even experience increased treatment toxicity (13,14). In contrast, patients with microsatellite stable (MSS)/proficient mismatch repair (pMMR) status should receive neoadjuvant chemotherapy (14,15). Thus, accurate preoperative assessment and monitoring of MSI/dMMR status are clinically essential.

Currently, MSI/dMMR testing based on preoperative biopsy is the standard procedure for newly diagnosed patients with EGJA (1). However, owing to the limited volume of biopsy samples and inherent tumor heterogeneity, preoperative biopsy results might differ from surgical pathology findings (16,17). Consequently, an adjunctive preoperative tool to supplement biopsy is needed. In the case of discordance between such a tool and the biopsy results, pathologists and clinicians could perform a reassessment, thereby ensuring a more reliable preoperative assessment of MSI/dMMR status in patients with EGJA.

In many centers, enhanced CT scanning is recommended by guidelines and is widely used for preoperative clinical staging and assessment of treatment efficacy in patients with EGJA (1). Compared with conventional CT, spectral CT provides multienergy virtual monoenergetic images (VMIs) and quantitative multiparameter data correlated with tissue composition, such as iodine density (ID) and effective atomic number (Z_eff_), thereby supplementing morphologic imaging with additional information (18,19). Derived from material decomposition, ID provides visualization and quantification of iodine within lesions, whereas Z_eff_ provides tissue characterization based on atomic numbers (19,20). These parameters have been used to predict pathophysiologic biomarkers, such as Ki-67 expression and TP53 status in patients with gastric cancer and MSI/dMMR status in patients with colorectal cancer (21–23). However, to our knowledge, no prior studies have investigated the feasibility of quantitative spectral CT parameters for the prediction of MSI/dMMR status in patients with EGJA, nor has their potential role as a supplement to preoperative biopsy been explored.

Therefore, we hypothesized that spectral CT could reflect the MSI/dMMR characteristics of EGJA. We aimed to establish a predictive model based on quantitative spectral CT parameters that, when used in conjunction with biopsy, could increase the accuracy of preoperative MSI/dMMR diagnosis in patients with EGJA.

## Materials and Methods

This multicenter study was approved by the institutional review boards of the First Affiliated Hospital of Fujian Medical University (Medical Research and Clinical Technology Application, Ethics Committee of First Affiliated Hospital of Fujian Medical University [2022] 334) and Zhongshan Hospital Xiamen University (xmzsyyky, no. 2022-082). Some patients were recruited retrospectively, and the requirement for informed consent was waived. Other participants were recruited prospectively, and all participants signed informed consent forms. This trial has been registered in the Chinese Clinical Trial Registry (nos. ChiCTR2500097335 and ChiCTR2500101639).

### Study Design and Sample

This study included patients with pathologically confirmed EGJA from two hospitals (center 1: the First Affiliated Hospital of Fujian Medical University and center 2: Zhongshan Hospital Xiamen University). Figure 1 shows the flow diagram of inclusion and exclusion. The inclusion criteria for the retrospective training, validation, and external test cohorts in this study were as follows: *(a)* underwent surgical pathology and was diagnosed with EGJA at clinical T1-4N0-3M0 according to the eighth edition of the *American Joint Committee on Cancer (AJCC) Cancer Staging Manual *(1), *(b)* underwent radical resection without neoadjuvant therapy, *(c)* underwent dual-phase contrast-enhanced spectral CT scanning of the chest, abdomen, and pelvis within 2 weeks before surgery, and *(d)* underwent immunohistochemical staining and polymerase chain reaction or next-generation sequencing for the diagnosis of MSI/dMMR. The exclusion criteria for the retrospective cohorts are detailed in Appendix S1.

**Figure 1:**
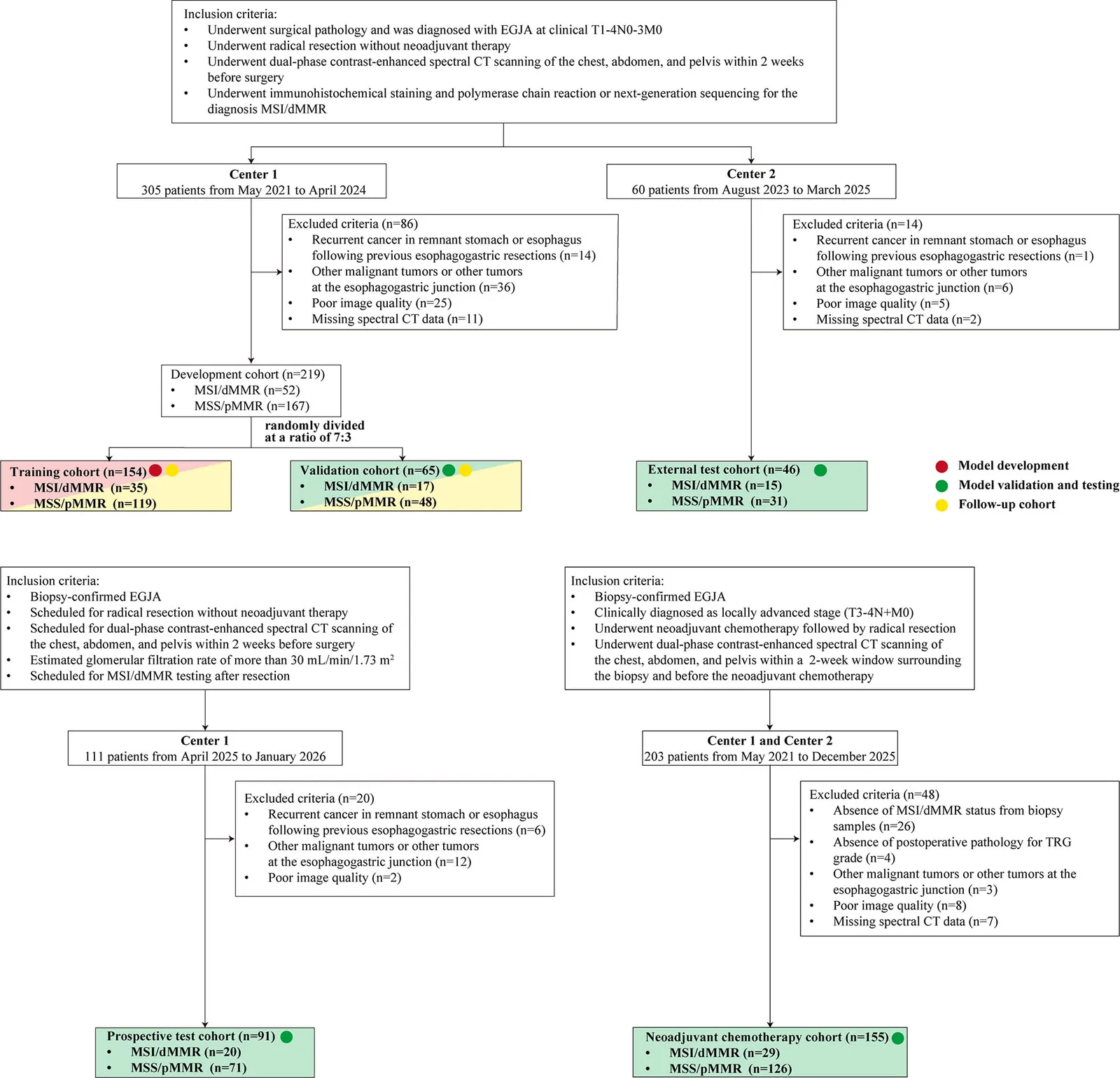
Flowchart of study sample inclusion and exclusion. Cohorts marked with red, green, and yellow circles represent the model development, model validation and testing, and follow-up cohorts, respectively. Center 1 = First Affiliated Hospital of Fujian Medical University, center 2 = Zhongshan Hospital Xiamen University, EGJA = esophagogastric junction adenocarcinoma, MSI/dMMR = microsatellite instability/deficient mismatch repair, MSS/pMMR = microsatellite stable/proficient mismatch repair, TRG = tumor regression grade.

Patients with biopsy-confirmed EGJA who met specific clinical and CT requirements were included in the neoadjuvant chemotherapy cohort and the prospective test cohort, which was specifically designed for the real-world validation of the models’ predictive accuracy. The detailed inclusion and exclusion criteria are provided in Appendix S1.

Between May 2021 and January 2026, a total of 511 patients from two centers were ultimately included in this study (Fig 1). The treatment regimens for the patients are detailed in Appendix S1. The patients in the training cohort and the validation cohort were followed up for survival analysis. The sample size estimation is provided in Appendix S1.

The primary end point of this study was to evaluate the performance of spectral CT in predicting MSI/dMMR status. The exploratory secondary end point was to assess the prognostic value of the nomogram regarding disease-free survival (DFS) in the retrospective training and validation cohorts. This study was performed in accordance with the Strengthening the Reporting of Observational Studies in Epidemiology (ie, STROBE) guidelines (24).

### Pathohistologic Evaluation

Surgical and biopsy samples from the esophagogastric junction were independently assessed for MSI/dMMR status by a pathologist (M.W., with 20 years of experience) using immunohistochemical staining and polymerase chain reaction or next-generation sequencing. The detailed procedures and the assessment of tumor regression grade in the neoadjuvant chemotherapy cohort are provided in Appendix S2.

### Clinicopathologic Data Collection

Clinicopathologic data included age, sex, carbohydrate antigen 19-9 level, carcinoembryonic antigen level, Siewert type, Lauren type, and histologic grade. Clinical T stage (cT stage) and clinical N stage (cN stage) were identified with contrast-enhanced CT with reference to the *AJCC Cancer Staging Manual* (1) and the Chinese clinical guidelines for diagnosis and treatment (25). Patients in the training and validation cohorts were seen regularly postoperatively to record follow-up data and DFS time. The follow-up protocols are provided in Appendix S3.

### Collection and Postprocessing of Spectral CT Data

CT scans for all patients at center 1 and center 2 were performed using a spectral CT scanner (IQon Spectral CT; Philips Healthcare). The CT scan and postprocessing procedures are detailed in Appendix S4.

### Image Analysis and Parameter Measurement

The spectral CT data were measured and analyzed using a postprocessing workstation (IntelliSpace Portal 10.0; Philips Healthcare). Data were measured independently by two radiologists (Y.W. and K.L., with 10 and 12 years of gastrointestinal radiology experience, respectively). For quantitative data, the average of the two observers’ results was used as the final result. For qualitative data, if disagreements arose during the evaluation process, a third radiologist (D.C., with 37 years of experience) was consulted to reach a final consensus.

In prior research, the VMI at 40 keV in the venous phase (VP) scan demonstrated optimal performance in terms of both subjective and objective evaluation indicators for gastric and esophageal cancer (26,27). Consequently, using the 40-keV VMI during the VP, we measured the longest tumor diameter on the axial plane and assessed the cT stage and cN stage. Quantitative spectral CT parameters were measured in the arterial phase and VP, including CT attenuation in VMIs, Z_eff_ value, and ID value. The definitions of cT stage, cN stage, and detailed protocols for spectral CT parameters are specified in Appendix S5.

### Model Development

In the training cohort, clinicopathologic variables and quantitative spectral CT parameters were initially considered as candidate predictors. Candidate clinicopathologic predictors included age, sex, carbohydrate antigen 19-9 level, carcinoembryonic antigen level, cT stage, cN stage, Siewert type, Lauren type, and histologic grade. Candidate spectral predictors comprised CT attenuation for VMIs at different energy levels during the arterial phase and VP, as well as Z_eff_, normalized Z_eff_, ID, and normalized ID (NID) values. To begin, univariable logistic regression analysis was applied to screen all candidate variables. Variables with *P* < .05 were retained. Collinearity diagnostics were performed to assess potential multicollinearity. Only predictors with a variance inflation factor less than 3 were selected for further modeling. Subsequently, multivariable logistic regression analysis was performed using a backward stepwise selection process based on the Akaike information criterion. This approach evaluated the independent spectral CT parameters, including CT attenuation in VMIs and NID, while adjusting for potential clinical confounding variables such as sex and cN stage. Finally, the selected predictors were combined to construct three models: clinical model, spectral CT model, and clinical spectral CT model developed by combining clinical predictors with quantitative spectral CT predictors, which was visualized as a nomogram. To explicitly evaluate the additive value of the nomogram when used in conjunction with preoperative biopsy, we constructed a combined model using biopsy status and nomogram results via multivariable logistic regression.

To evaluate the feasibility of substituting spectral CT parameters with nonspectral CT measurement, we simulated 120-kVp images during the VP using 70-keV VMIs that approximate 120-kVp images (20). The detailed construction methods for four 120-kVp–like models are described in Appendix S6.

Through mathematical modeling, the regression coefficients and intercepts derived from multivariable logistic regression were used to formulate the linear predictive equations for calculating each model score. The MSI/dMMR predictive index was calculated as follows: Index = 1/[1 + exp (−model score)], where *exp *represents the exponential function. Given the inherent class imbalance resulting from the relatively low prevalence of MSI/dMMR in the study population, the optimal cutoff threshold for the predictive index was determined by maximizing the Youden index. To ensure generalizability, we performed bootstrap validation with 1000 resamples for all models across the five cohorts.

### Statistical Analysis

The data were analyzed using MedCalc (version 20.0.22; MedCalc Software) and R software (version 4.3.2; R Foundation, *https://www.R-project.org*) (Y.W. and K.L.). A priori power analysis was conducted to determine the sample size for this study. Statistical analysis details are provided in Appendix S7. Briefly, the area under the receiver operating characteristic curve (AUC) and 95% CI were evaluated. Survival curves for DFS in follow-up patients were generated using the Kaplan-Meier method and compared using the log-rank test. A two-tailed *P* < .05 was considered to indicate statistical significance.

## Results

### Clinicopathologic Characteristics

In this study, we recruited 511 patients with EGJA (median age, 67 years [IQR, 60–73 years]; 333 male), who were divided into five cohorts (Fig 1). Detailed information on patient inclusion and exclusion criteria is provided in Appendix S8. In total, 154 patients (median age, 68 years [IQR, 62–74 years]; 92 male) were included in the training cohort, 65 patients (median age, 68 years [IQR, 62–72 years]; 43 male) were included in the validation cohort, 46 patients (median age, 70 years [IQR, 60–75 years]; 33 male) were included in the external test cohort, 91 patients (median age, 72 years [IQR, 61–75 years]; 53 male) were included in the prospective test cohort, and 155 patients (median age, 63 years [IQR, 57–69 years]; 112 male) were included in the neoadjuvant chemotherapy cohort. According to the pathologic results of the surgical resection samples, the proportion of patients with MSI/dMMR in the training, validation, external test, and prospective test cohorts was 23% (35 of 154), 26% (17 of 65), 33% (15 of 46), and 22% (20 of 91), respectively. Based on the preoperative biopsy findings, the proportion of patients with MSI/dMMR in the neoadjuvant chemotherapy cohort was 19% (29 of 155). Patient characteristics are summarized in Table 1 and Table S1.

**Table 1:** Clinicopathologic Variables of Patients among Different Cohorts

Variables	Training Cohort (*n* = 154)		Validation Cohort (*n* = 65)		External Test Cohort (*n* = 46)		Prospective Test Cohort (*n* = 91)		Neoadjuvant Chemotherapy Cohort (*n* = 155)	
	MSI/dMMR (*n* = 35)	MSS/pMMR (*n* = 119)	MSI/dMMR (*n* = 17)	MSS/pMMR (*n* = 48)	MSI/dMMR (*n* = 15)	MSS/pMMR (*n* = 31)	MSI/dMMR (*n* = 20)	MSS/pMMR (*n* = 71)	MSI/dMMR (*n* = 29)	MSS/pMMR (*n* = 126)
Age (y)	68 (63–76)	68 (61–74)	68 (65–71)	68 (61–73)	73 (70–78)	65 (59–75)	73 (61–77)	71 (62–74)	63 (58–70)	63 (56–69)
Sex										
Male	13 (37)	79 (66)	10 (59)	33 (69)	7 (47)	26 (84)	13 (65)	40 (56)	6 (21)	106 (84)
Female	22 (63)	40 (34)	7 (41)	15 (31)	8 (53)	5 (16)	7 (35)	31 (44)	23 (79)	20 (16)
CA 19-9 level										
Normal	25 (71)	85 (72)	12 (71)	33 (69)	11 (73)	24 (77)	15 (75)	54 (76)	21 (73)	91 (72)
Elevated	8 (23)	24 (20)	3 (17)	13 (27)	4 (27)	7 (23)	4 (20)	15 (21)	5 (17)	27 (22)
Missing	2 (6)	10 (8)	2 (12)	2 (4)	0 (0)	0 (0)	1 (5)	2 (3)	3 (10)	8 (6)
CEA level										
Normal	21 (60)	88 (74)	10 (59)	33 (69)	12 (80)	25 (81)	12 (60)	53 (75)	24 (83)	91 (72)
Elevated	12 (34)	21 (18)	5 (29)	13 (27)	3 (20)	6 (19)	7 (35)	16 (22)	5 (17)	26 (21)
Missing	2 (6)	10 (8)	2 (12)	2 (4)	0 (0)	0 (0)	1 (5)	2 (3)	0 (0)	9 (7)
cT stage										
T1–2	14 (40)	30 (25)	5 (29)	12 (25)	5 (33)	6 (19)	5 (25)	15 (21)	0 (0)	0 (0)
T3	10 (29)	45 (38)	7 (42)	24 (50)	3 (20)	12 (39)	4 (20)	30 (42)	15 (52)	57 (45)
T4	11 (31)	44 (37)	5 (29)	12 (25)	7 (47)	13 (42)	11 (55)	26 (37)	14 (48)	69 (55)
cN stage										
N0	20 (57)	25 (21)	12 (71)	33 (69)	11 (73)	14 (45)	16 (80)	25 (35)	20 (69)	37 (29)
N1–3	15 (43)	94 (79)	5 (29)	15 (31)	4 (27)	17 (55)	4 (20)	46 (65)	9 (31)	89 (71)
Biopsy result										
pMMR	2 (6)	50 (42)	1 (6)	15 (31)	1 (7)	9 (29)	1 (5)	16 (22)	NA	NA
dMMR	14 (40)	4 (3)	5 (29)	2 (4)	4 (26)	0 (0)	3 (15)	2 (3)	NA	NA
MSI/dMMR	14 (40)	5 (4)	7 (41)	4 (8)	7 (47)	2 (6)	11 (55)	4 (6)	29 (100)	NA
MSS/pMMR	3 (8)	46 (39)	1 (6)	22 (46)	1 (7)	17 (55)	3 (15)	46 (65)	NA	126 (100)
Missing	2 (6)	14 (12)	3 (18)	5 (11)	2 (13)	3 (10)	2 (10)	3 (4)	NA	NA

Note.—Data are reported as medians, with IQRs in parentheses, or as numbers of patients, with percentages in parentheses. The reference range for carbohydrate antigen 19-9 (CA 19-9) level is 0–34 U/mL, and that for carcinoembryonic antigen (CEA) is 0–4.7 ng/mL. For the neoadjuvant chemotherapy cohort, the classification into microsatellite instability/deficient mismatch repair (MSI/dMMR) and microsatellite stable/proficient mismatch repair (MSS/pMMR) was based on the pathologic results from preoperative biopsy specimens. cN stage = clinical N stage, cT stage = clinical T stage, NA = not applicable.

### Interobserver Agreement

The intraclass correlation coefficients for the maximum diameter and spectral CT parameters measured by the two observers were excellent (range, 0.85 [95% CI: 0.82, 0.87] to 0.96 [95% CI: 0.95, 0.97]) (Table S2). The two observers also reported strong agreement for the cT and cN stages, with κ values of 0.84 (95% CI: 0.80, 0.88) and 0.86 (95% CI: 0.82, 0.91), respectively (Table S3). Overall, these findings indicated a high reliability of the measurements.

### Clinical Model Performance

In the training cohort, univariable and multivariable logistic regression analyses revealed cN stage and sex as independent predictors for MSI/dMMR (Table 2). The clinical model was constructed using the derived regression coefficients: clinical model score = −2.41 + [1.21 × sex (female)] + [1.61 × cN stage (N0)] (Table S4). In this model, female sex or cN0 stage was assigned a value of 1, and male sex or cN1–3 stage was assigned a value of 0. Maximizing the Youden index yielded an ideal index threshold of 0.16 for predicting MSI/dMMR. The AUC values in the training, validation, external test, prospective test, and neoadjuvant chemotherapy cohorts were 0.75 (95% CI: 0.66, 0.84), 0.72 (95% CI: 0.59, 0.85), 0.72 (95% CI: 0.56, 0.88), 0.69 (95% CI: 0.57, 0.81), and 0.72 (95% CI: 0.62, 0.81), respectively (Table 3).

**Table 2:** Univariable and Multivariable Logistic Regression Analyses of Clinicopathologic Characteristics in Training Cohort

Variables	Univariable Analysis		Multivariable Analysis	
	Odds Ratio	*P* Value	Odds Ratio	*P* Value
Age	1.00 (0.96, 1.05)	.85		
Sex (female vs male)	3.34 (1.53, 7.32)	.003	3.58 (1.53, 8.42)	.003*
CA 19-9 level (elevated vs normal)	1.22 (0.59, 2.52)	.59		
CEA level (elevated vs normal)	2.04 (0.98, 4.24)	.06		
cT stage				
T1–2	Ref.			
T3	0.48 (0.19, 1.21)	.12		
T4	0.54 (0.21, 1.34)	.18		
cN stage (cN0 vs cN1–3)	5.01 (2.25, 11.18)	<.001	4.73 (1.98, 11.25)	<.001*
Siewert type				
I	Ref			
II	0.68 (0.24, 1.93)	.47		
III	2.00 (0.83, 4.83)	.12		
Lauren type				
Intestinal	Ref			Ref
Mixed	0.32 (0.12, 0.89)	.03	0.35 (0.12, 1.08)	.07
Diffuse	0.88 (0.37, 2.09)	.77	1.10 (0.41, 2.95)	.86
Histologic grade				
Poor	Ref			
Moderate	0.98 (0.42, 2.29)	.97		
Well	2.62 (0.90, 7.63)	.08		

Note.—Data in parentheses are 95% CIs. Odds ratios were derived from univariable and multivariable logistic regression analyses. For binary variables, the odds ratios reflect second category likelihood relative to the first. CA 19-9 = carbohydrate antigen 19-9, CEA = carcinoembryonic antigen, cN stage = clinical N stage, cT stage = clinical T stage, Ref = reference standard.

* Variables ultimately selected through multivariable logistic regression analysis (*P* < .05).

**Table 3:** Performance of Clinical, Spectral CT, and Nomogram Models for Predicting Microsatellite Instability/Deficient Mismatch Repair Status

Models	AUC	Accuracy (%)	Sensitivity (%)	Specificity (%)	PPV (%)	NPV (%)
Clinical						
Training cohort	0.75 (0.66, 0.84)	60 (93/154)	86 (30/35)	53 (63/119)	35 (30/86)	93 (63/68)
Validation cohort	0.72 (0.59, 0.85)	62 (40/65)	88 (15/17)	52 (25/48)	39 (15/38)	93 (25/27)
External test cohort	0.72 (0.56, 0.88)	76 (35/46)	47 (7/15)	90 (28/31)	70 (7/10)	78 (28/36)
Prospective test cohort	0.69 (0.57, 0.81)	68 (62/91)	80 (16/20)	65 (46/71)	39 (16/41)	92 (46/50)
Neoadjuvant chemotherapy cohort	0.72 (0.62, 0.81)	70 (109/155)	69 (20/29)	71 (89/126)	35 (20/57)	91 (89/98)
Spectral CT						
Training cohort	0.81 (0.72, 0.89)	79 (122/154)	71 (25/35)	82 (97/119)	53 (25/47)	91 (97/107)
Validation cohort	0.83 (0.71, 0.95)	85 (55/65)	71 (12/17)	90 (43/48)	71 (12/17)	90 (43/48)
External test cohort	0.81 (0.68, 0.95)	78 (36/46)	93 (14/15)	71 (22/31)	61 (14/23)	96 (22/23)
Prospective test cohort	0.84 (0.75, 0.94)	71 (65/91)	90 (18/20)	66 (47/71)	43 (18/42)	96 (47/49)
Neoadjuvant chemotherapy cohort	0.81 (0.70, 0.92)	90 (139/155)	59 (17/29)	97 (122/126)	81 (17/21)	91 (122/134)
Nomogram						
Training cohort	0.87 (0.81, 0.93)	76 (117/154)	89 (31/35)	72 (86/119)	48 (31/64)	96 (86/90)
Validation cohort	0.87 (0.78, 0.96)	75 (49/65)	94 (16/17)	69 (33/48)	52 (16/31)	97 (33/34)
External test cohort	0.91 (0.83, 0.99)	78 (36/46)	100 (15/15)	68 (21/31)	60 (15/25)	100 (21/21)
Prospective test cohort	0.89 (0.80, 0.98)	90 (82/91)	70 (14/20)	96 (68/71)	82 (14/17)	92 (68/74)
Neoadjuvant chemotherapy cohort	0.86 (0.76, 0.96)	86 (133/155)	76 (22/29)	88 (111/126)	59 (22/37)	94 (111/118)

Note.—For areas under the receiver operating characteristic curve (AUCs), data in parentheses are 95% CIs. For accuracy, sensitivity, specificity, positive predictive value (PPV), and negative predictive value (NPV), values in parentheses are numerators and denominators.

### Spectral CT Model Performance

In the training cohort, CT attenuation at a VMI of 40 keV and NID in the VP were independent predictors of MSI/dMMR, as demonstrated by univariable and multivariable logistic regression analyses (Table S5). Collinearity diagnostics revealed that the variance inflation factors for both predictors were 1.00, indicating no collinearity. The spectral CT model was calculated as follows: spectral CT model score = 8.96 – (0.02 × CT_40keV_VP_) – (9.32 × NID_VP_), where CT_40keV_VP_ is CT attenuation in 40-keV VMIs in the VP and NID_VP_ is NID in the VP (Table S4). The optimal predictive index threshold for MSI/dMMR status was 0.26, which was determined by maximizing the Youden index. Across all five cohorts, the CT_40keV_ attenuation and NID values during the arterial phase and VP were lower in patients with MSI/dMMR status than in those with MSS/pMMR status (*P* < .05 for all, Table S6).

The predictive performance of the spectral CT model across the five cohorts is summarized in Table 3. In the prospective test cohort, the AUC reached 0.84 (95% CI: 0.75, 0.94). The spectral CT model increased the AUC compared with the clinical model (ΔAUCs, 0.06, 0.11, 0.09, 0.15, and 0.09; *P* = .42, *P* = .22, *P* = .45, *P* = .08, and *P* = .28, respectively). Detailed results comparing the AUCs of the two models are presented in Table S7 and Appendix S9. The diagnostic concordance between the preoperative biopsy results and the spectral CT predictions was moderate, with κ values ranging from 0.26 (95% CI: 0.08, 0.44) to 0.62 (95% CI: 0.45, 0.79) (Fig S1).

### Nomogram Performance

By integrating clinical and spectral CT predictors, a clinical spectral CT model was constructed and visualized as a nomogram (Fig 2A). Based on the regression coefficients (Table S4), the clinical spectral CT model score was obtained as follows: clinical spectral CT model score = 8.17 + [1.07 × sex (female)] + [1.81 × cN stage (N0)] − (0.02 × CT_40keV_VP_) – (10.33 × NID_VP_). The optimal cutoff threshold for the MSI/dMMR predictive index was 0.17, corresponding to 100 total points. Patients for whom the index exceeded this threshold were predicted to have MSI/dMMR status.

**Figure 2:**
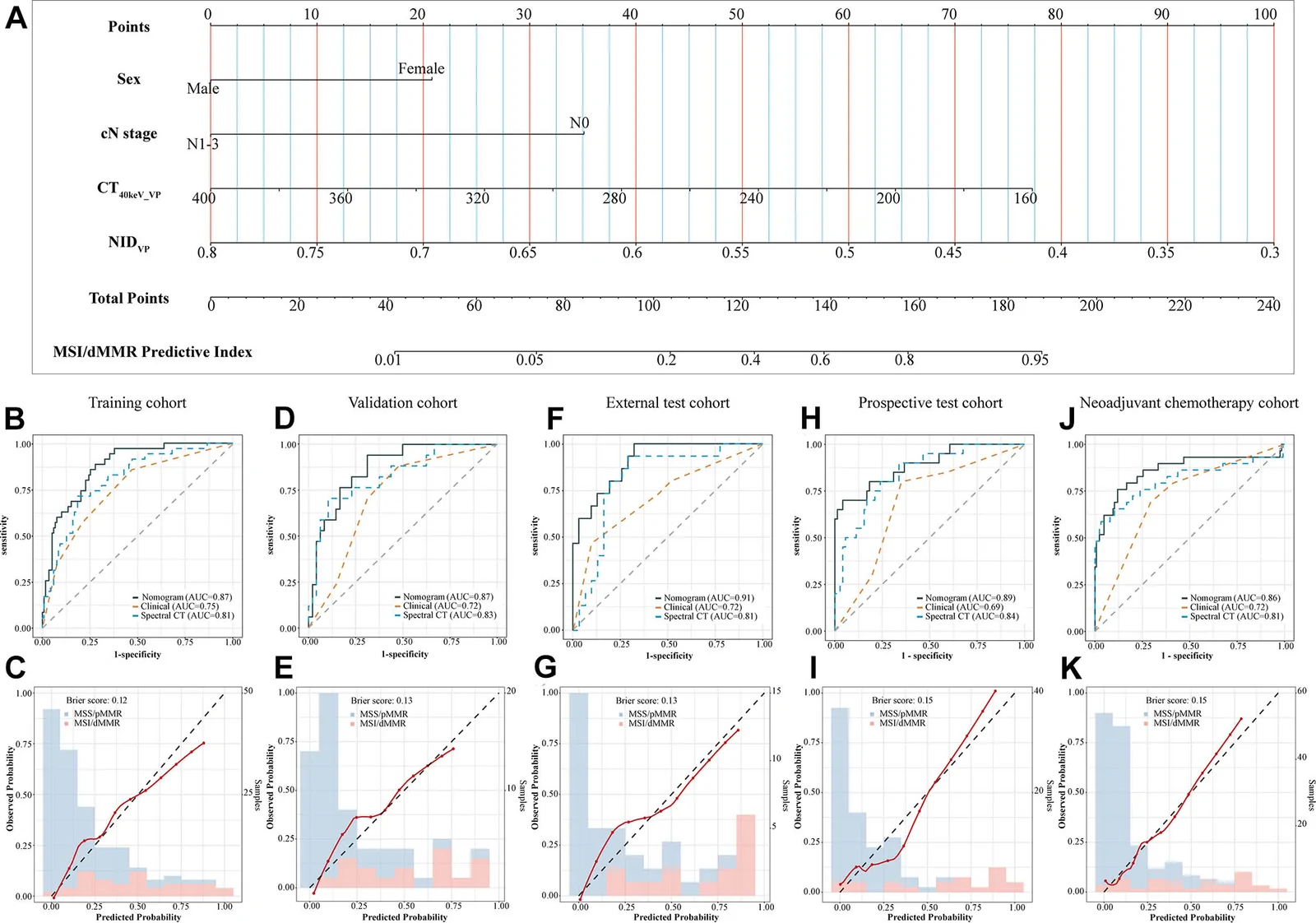
The nomogram and the predictive performance for predicting microsatellite instability/deficient mismatch repair (MSI/dMMR). **(A)** The nomogram shows the MSI/dMMR predictive index. The areas under the receiver operating characteristic curves (AUCs) for the clinical model, the spectral CT model, and the nomogram are shown, as well as the calibration curves of the nomogram based on the **(B, C)** training cohort, **(D, E)** validation cohort, **(F, G)** external test cohort, **(H, I)** prospective test cohort, and **(J, K)** neoadjuvant chemotherapy cohort. The red and blue areas represent the number of patients with MSI/dMMR and patients with microsatellite stable/proficient mismatch repair (MSS/pMMR), respectively. cN stage = clinical N stage, CT_40keV_VP _= CT attenuation in 40-keV virtual monoenergetic images in venous phase, ID = iodine density, NID = normalized ID, NID_VP_ = ID_tumor_/ID_aorta_ in venous phase.

The Akaike information criterion values were 146.757 for the clinical model, 138.211 for the spectral CT model, and 121.891 for the nomogram, indicating that the nomogram provided better predictive performance with the lowest risk of overfitting. As depicted in Figure 2B–2K and Table 3, the nomogram demonstrated satisfactory predictive performance across all cohorts, with AUCs ranging from 0.86 (95% CI: 0.76, 0.96) to 0.91 (95% CI: 0.83, 0.99). Table S7 and Appendix S10 present detailed results demonstrating that the nomogram achieved higher efficacy than the clinical model and the spectral CT model. In all the cohorts, the predicted MSI/dMMR probabilities strongly agreed with the actual probabilities (Brier score range, 0.12–0.15; Fig 2), indicating that the nomogram’s predictions were reliable for clinical application.

Moreover, comprehensive subgroup analyses were performed on the entire study population and across all five cohorts, stratified by age, sex, carbohydrate antigen 19-9 level, carcinoembryonic antigen level, cT stage, cN stage, Siewert type, Lauren type, and histologic grade, resulting in a total of 130 distinct subgroups. The nomogram demonstrated robust predictive performance, with 92% (120 of 130) of the subgroups showing AUCs exceeding 0.80 (Fig S2, Appendix S10). We also evaluated the predictive performance of the 120-kVp–like model across the five cohorts (Table S8, Fig S3). As shown in Table S9, the AUCs of the 120-kVp–like model was lower than that of the nomogram (ΔAUCs, 0.10, 0.04, 0.11, 0.11, and 0.12; *P* = .02, *P* = .52, *P* = .06, *P* = .01, and *P* = .10, respectively).

We also assessed the relative contributions of individual variables to MSI/dMMR prediction. Across the three cohorts, regardless of which preoperative indicator dominated the clinical model, the nomogram score became the primary predictive variable after its inclusion (Fig 3). Furthermore, pairwise concordance was systematically assessed (Fig S1). The dMMR status predicted by the nomogram demonstrated moderate to high consistency with the biopsy results (κ value range, 0.46–0.72) and surgical pathology (κ value range, 0.51–0.78), thereby validating the reliability of our preoperative diagnostic model.

**Figure 3:**
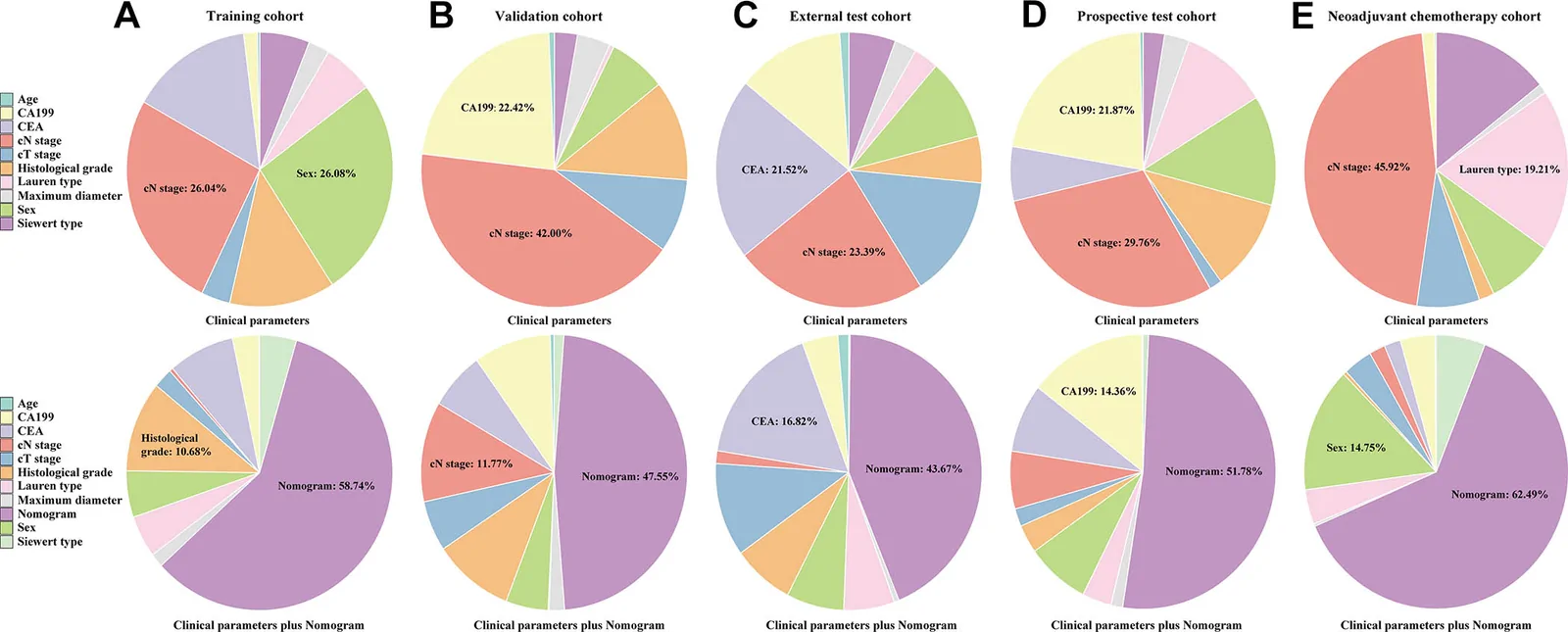
Relative contributions of different variables. The relative contributions of clinicopathologic indicators and the nomogram to the prediction results in the **(A)** training cohort, **(B)** validation cohort, **(C)** external test cohort, **(D)** prospective test cohort, and **(E)** neoadjuvant chemotherapy cohort according to the χ^2^ proportion test. CA199 = carbohydrate antigen 19-9, CEA = carcinoembryonic antigen, cN stage = clinical N stage, cT stage = clinical T stage.

### Additional Predictive Value of the Nomogram

We constructed confusion matrices to compare the nomogram predictions with the biopsy results across the training, validation, external test, and prospective test cohorts, using surgical pathology as the reference standard. The analysis included patients with complete and available preoperative CT images, biopsy results, and surgical pathology results. We observed some differences between the preoperative biopsy and surgical pathology results (Fig 4). Notably, in the training and validation cohorts, respectively, the nomogram correctly reclassified 1.45% (two of 138) and 3.51% (two of 57) of patients with biopsy-based pMMR and 1.45% (two of 138) and 5.26% (three of 57) of the patients diagnosed with dMMR. In the external test cohort, the nomogram successfully identified all the patients misclassified by biopsy. Within the prospective cohort, the nomogram accurately detected 4.65% (four of 86) of the patients with false-positive biopsy results. We also observed instances of incorrect reclassification by the nomogram. The proportion of true-negative biopsy findings reclassified as nomogram false-positive findings ranged from 0% (0 of 86) to 17.07% (seven of 41). The proportion of true-positive biopsy findings reclassified as false-negative nomogram findings ranged from 0% (0 of 138) to 2.33% (two of 86).

**Figure 4:**
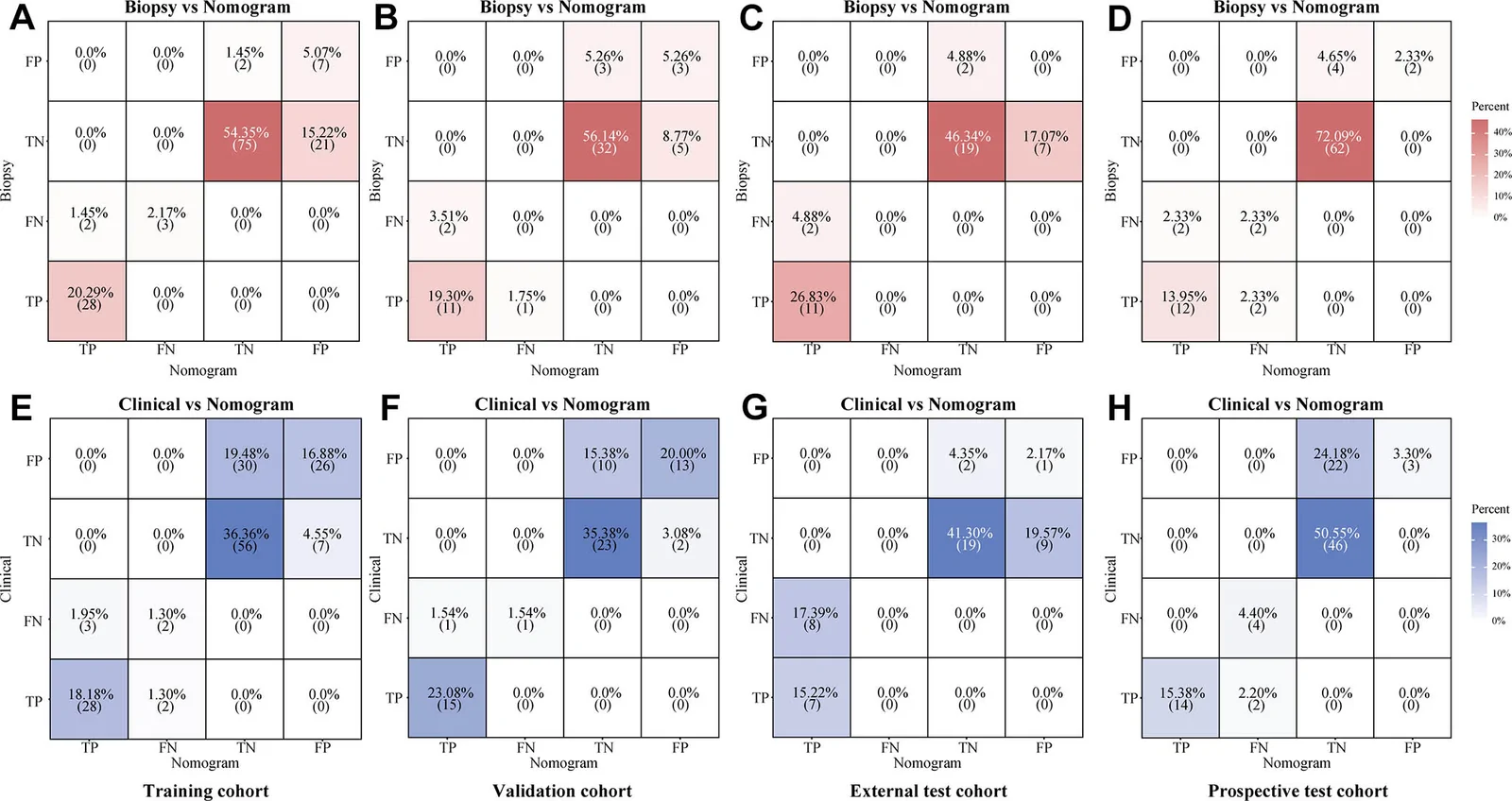
Confusion matrices for microsatellite instability/deficient mismatch repair status across the four cohorts. The top row shows a comparison of the diagnostic performance of the biopsy results versus the nomogram predictions, with surgical pathology used as the reference standard, across the **(A)** training, **(B)** validation, **(C)** external test, and **(D)** prospective test cohorts. The bottom row illustrates diagnostic discrepancies between the nomogram and the clinical model relative to the surgical reference standard across the **(E)** training, **(F)** validation, **(G)** external test, and **(H)** prospective test cohorts. Each cell displays the proportion and number of patients. FN = false negative, FP = false positive, TN = true negative, TP = true positive.

To illustrate the supplementary value of the nomogram, particularly its role as an adjunctive cross-reference tool to support preoperative biopsy findings, Figures S4 and S5 present two patients with false-negative and false-positive biopsy results, respectively. In one retrospective patient, endoscopic biopsy findings indicated an MSS/pMMR status, whereas the nomogram predicted an MSI/dMMR status; surgical pathology findings corroborated the status as MSI/dMMR. In one prospective patient, the initial biopsy findings suggested an MSI/dMMR status, but the nomogram predicted an MSS/pMMR status. Following reevaluation of the samples by the clinician and pathologist, the diagnosis was revised to MSS/pMMR, which was ultimately validated with surgical pathology.

In addition, to clarify the value of the nomogram as an adjunct to biopsy, we combined biopsy status with nomogram output to construct a biopsy-nomogram model. Across the training, validation, external test, and prospective test cohorts, the AUC values for the biopsy-nomogram model ranged from 0.92 (95% CI: 0.84, 1.00) to 0.98 (95% CI: 0.95, 1.00) (Table S10). We compared the predictive performance of biopsy alone with that of the biopsy-nomogram model. The results showed that the AUC of the biopsy-nomogram model was consistently higher than that of biopsy alone (Table S11). In the external test cohort, the AUC increased from 0.89 for biopsy alone to 0.98 for the biopsy-nomogram model (*P* = .048). Furthermore, the ability of the nomogram to correct misclassifications by the clinical model was robust (Fig 4). In the training cohort, the nomogram identified three patients missed by the clinical model and 30 misclassified patients, yielding a correction rate of 21.43% (33 of 154). The correction rate was 16.92% (11 of 65) in the validation cohort, 21.74% (10 of 46) in the external test cohort, and 24.18% (22 of 91) in the prospective test cohort. As examples, two representative patients are presented in Figure 5. The nomogram correctly identified one false-negative and one false-positive patient misclassified by the clinical model, aligning with the surgical pathology results. These results demonstrate the capacity of the nomogram to correct clinical classification errors.

**Figure 5:**
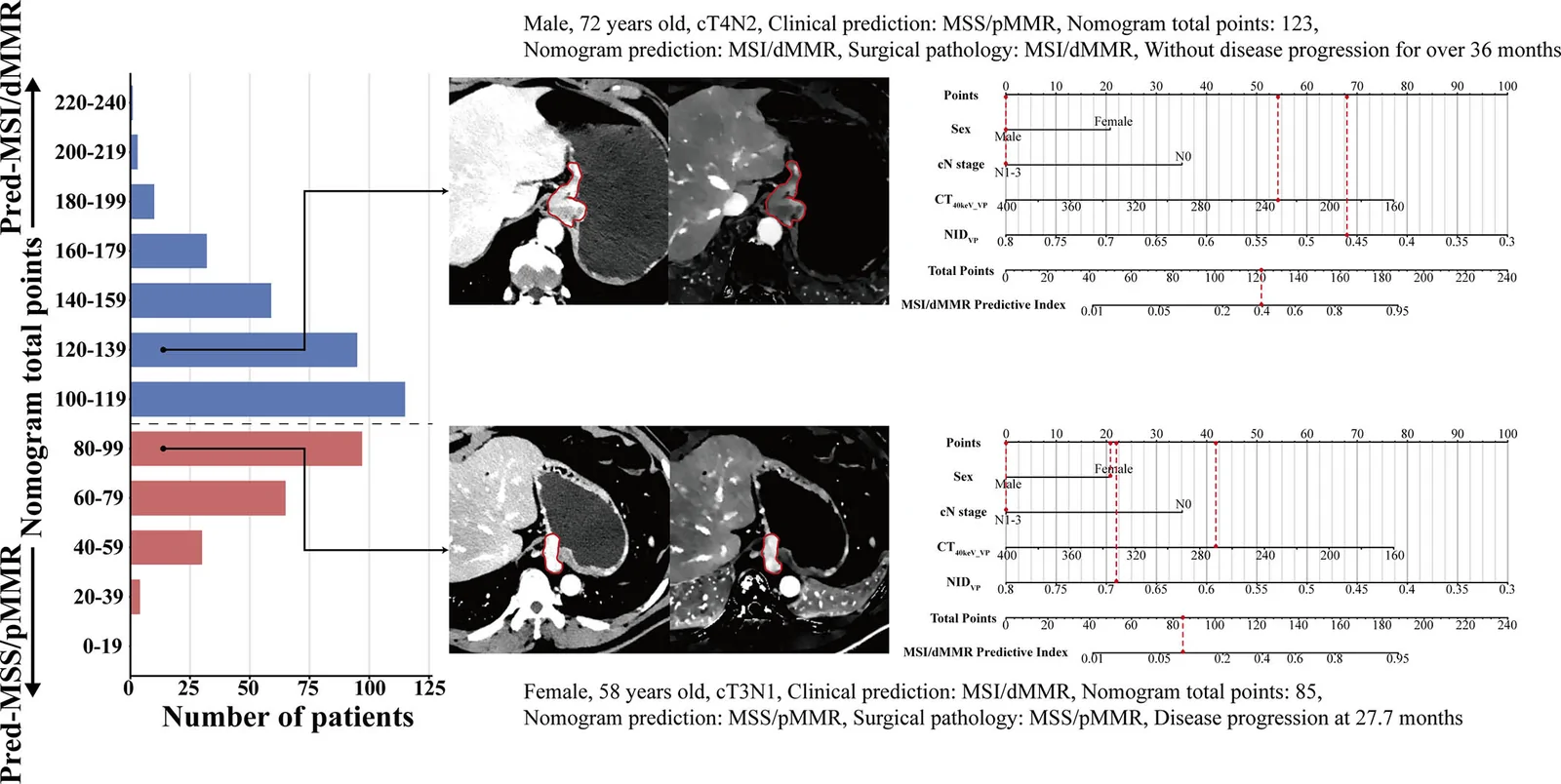
Patients misclassified by the clinical model corrected by the nomogram. A histogram of the nomogram total points distribution is shown on the left. The red and blue bars represent the numbers of patients with microsatellite stable/proficient mismatch repair (MSS/pMMR) and microsatellite instability/deficient mismatch repair (MSI/dMMR), respectively. The data for and images in two patients are displayed on the right. For both patients, the images display axial contrast-enhanced spectral CT scans, with the left panel showing a 40-keV virtual monoenergetic image and the right panel showing an iodine density image during venous phase. The tumor regions are delineated by red outlines. Top right: Patient 1, a 72-year-old male patient with cT4N2, CT attenuation in 40-keV virtual monoenergetic images in venous phase (CT_40keV_VP_) of 231.50 HU, and normalized iodine density in the venous phase (NID_VP_) of 0.46, was clinically predicted as having an MSS/pMMR status, whereas the nomogram prediction (Pred) and pathologic results revealed MSI/dMMR status. Bottom right: Patient 2, a 58-year-old female patient with cT3N1, CT_40keV_VP_ of 270.10 HU, and NID_VP_ of 0.69 was clinically classified as having an MSI/dMMR status, whereas the nomogram prediction and pathologic results revealed MSS/pMMR status. cN stage = clinical N stage, cT = clinical T stage.

### Nomogram Performance for Prognosis

The median follow-up was 27.10 months in the training cohort and 13.60 months in the validation cohort. Based on the nomogram cutoff, the patients were divided into predicted MSI/dMMR and predicted MSS/pMMR groups. Kaplan-Meier survival curves indicated that the nomogram risk-stratified patients with EGJA (Fig 6). In both cohorts, DFS differed between MSI/dMMR and MSS/pMMR groups classified by the reference standard and the nomogram (training cohort, *P* < .001 for both; validation cohort, *P* = .024 and .003, respectively). Comprehensive median DFS values are provided in Appendix S11. The Schoenfeld residuals test showed that all *P* values were greater than .05. Univariable and multivariable Cox proportional hazards regression analyses confirmed that the nomogram score was a strong independent predictor of DFS in patients with EGJA (Table 4).

**Figure 6:**
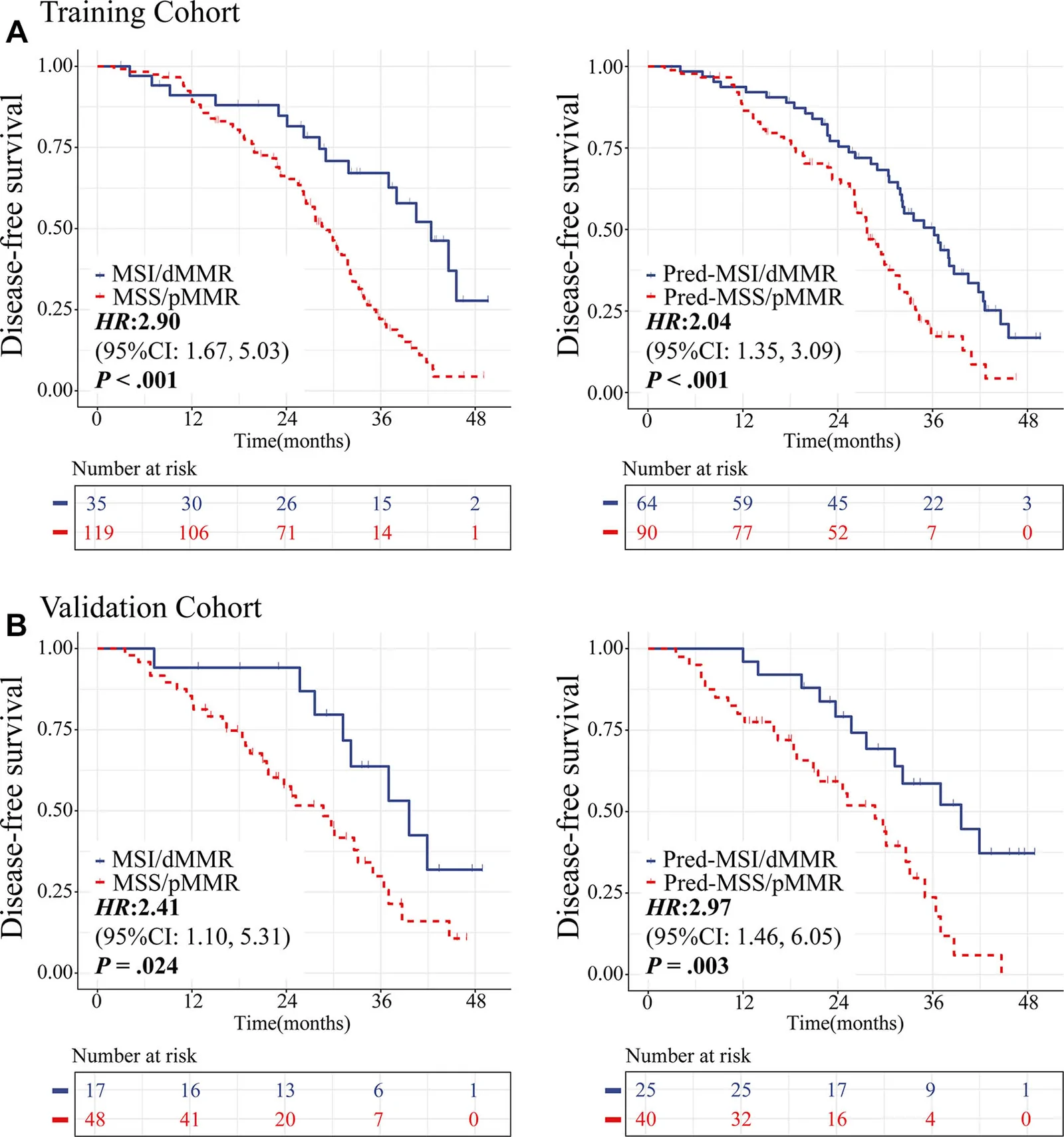
Kaplan-Meier survival curves related to the nomogram. Kaplan-Meier survival curves for **(A)** the training cohort and **(B)** the validation cohort. (Left) Curves based on the surgical reference standard labels and (right) those based on the nomogram prediction labels (Pred). HR = hazard ratio, MSI/dMMR = microsatellite instability/deficient mismatch repair, MSS/pMMR = microsatellite stable/proficient mismatch repair.

**Table 4:** Cox Regression Analysis of Models for Disease-Free Survival

Variables	Training Cohort (*n* = 154)				Validation Cohort (*n* = 65)			
	Univariable Analysis		Multivariable Analysis		Univariable Analysis		Multivariable Analysis	
	Hazard Ratio	*P* Value	Hazard Ratio	*P* Value	Hazard Ratio	*P* Value	Hazard Ratio	*P* Value
Nomogram score	0.82 (0.73, 0.92)	<.001	0.83 (0.74, 0.93)	<.001	0.79 (0.66, 0.94)	.009	0.79 (0.66, 0.94)	.009
Age	0.99 (0.96, 1.01)	.19			1.00 (0.97, 1.03)	.87		
Sex (female vs male)	0.67 (0.45, 1.00)	.048			0.53 (0.25, 1.11)	.09		
CA 19-9 level (elevated vs normal)	1.38 (0.87, 2.19)	.17			1.28 (0.62, 2.63)	.51		
CEA level (elevated vs normal)	1.02 (0.65, 1.60)	.93			0.93 (0.45, 1.92)	.85		
Siewert type								
I	Ref				Ref			
II	1.24 (0.77, 1.98)	.37			1.21 (0.54, 2.72)	.64		
III	0.88 (0.55, 1.41)	.61			0.88 (0.39, 1.99)	.76		
cT stage								
T1–2	Ref				Ref			
T3	1.83 (1.12, 2.98)	.02	1.64 (1.00, 2.69)	.049	2.71 (1.10, 6.69)	.03		
T4	1.28 (0.79, 2.09)	.32	1.12 (0.68, 1.83)	.65	2.36 (0.85, 6.52)	.10		
cN stage (cN0 vs cN1–3)	0.52 (0.33, 0.82)	.005			0.71 (0.37, 1.36)	.30		
Lauren type								
Intestinal	Ref				Ref			
Mixed	0.87 (0.55, 1.38)	.56			0.73 (0.36, 1.49)	.39		
Diffuse	0.85 (0.53, 1.36)	.50			0.62 (0.26, 1.47)	.28		
Histologic grade								
Poor	Ref				Ref			
Moderate	1.05 (0.69, 1.58)	.83			1.52 (0.78, 3.00)	.22		
Well	1.31 (0.71, 2.44)	.39			1.04 (0.38, 2.85)	.93		
Maximum diameter	1.00 (0.91, 1.09)	.93			0.93 (0.78, 1.10)	.40		

Note.—Data in parentheses are 95% CIs. Hazard ratios were derived from univariable and multivariable Cox proportional hazards regression analyses. For binary variables, the hazard ratios reflect second category likelihood relative to the first. Maximum diameter was the longest tumor diameter measured on the axial plane in the 40-keV virtual monoenergetic image during the venous phase. To prevent multicollinearity and overfitting, a backward stepwise selection method based on the Akaike information criterion was used to construct the optimal multivariable model. CA 19-9 = carbohydrate antigen 19-9, CEA = carcinoembryonic antigen, cN stage = clinical N stage, cT stage = clinical T stage, Ref = reference standard.

Disease progression within 2 years in the patients with high-risk MSS/pMMR EGJA was compared between the nomogram and the clinical model (Fig 7). In the training cohort, the disease progression detection rate increased from 36.76% based on the clinical model to 42.22% based on the nomogram. In the validation cohort, that rate increased from 40.74% (clinical model) to 60.00% (nomogram). Additionally, the nomogram outperformed the clinical model in excluding the patients with low-risk MSI/dMMR EGJA without likely 2-year disease progression. In the training and validation cohorts, respectively, the detection rates were 32.56% (clinical model) versus 29.69% (nomogram) and 47.37% (clinical model) versus 32.00% (nomogram). We also evaluated the predictive performance of the nomogram relative to biopsy for assessing the response to neoadjuvant chemotherapy (Fig S6). The nomogram achieved an AUC of 0.71, outperforming biopsy assessment (AUC = 0.61; DeLong test, *P* = .01), thereby confirming the prognostic predictive value of the nomogram.

**Figure 7:**
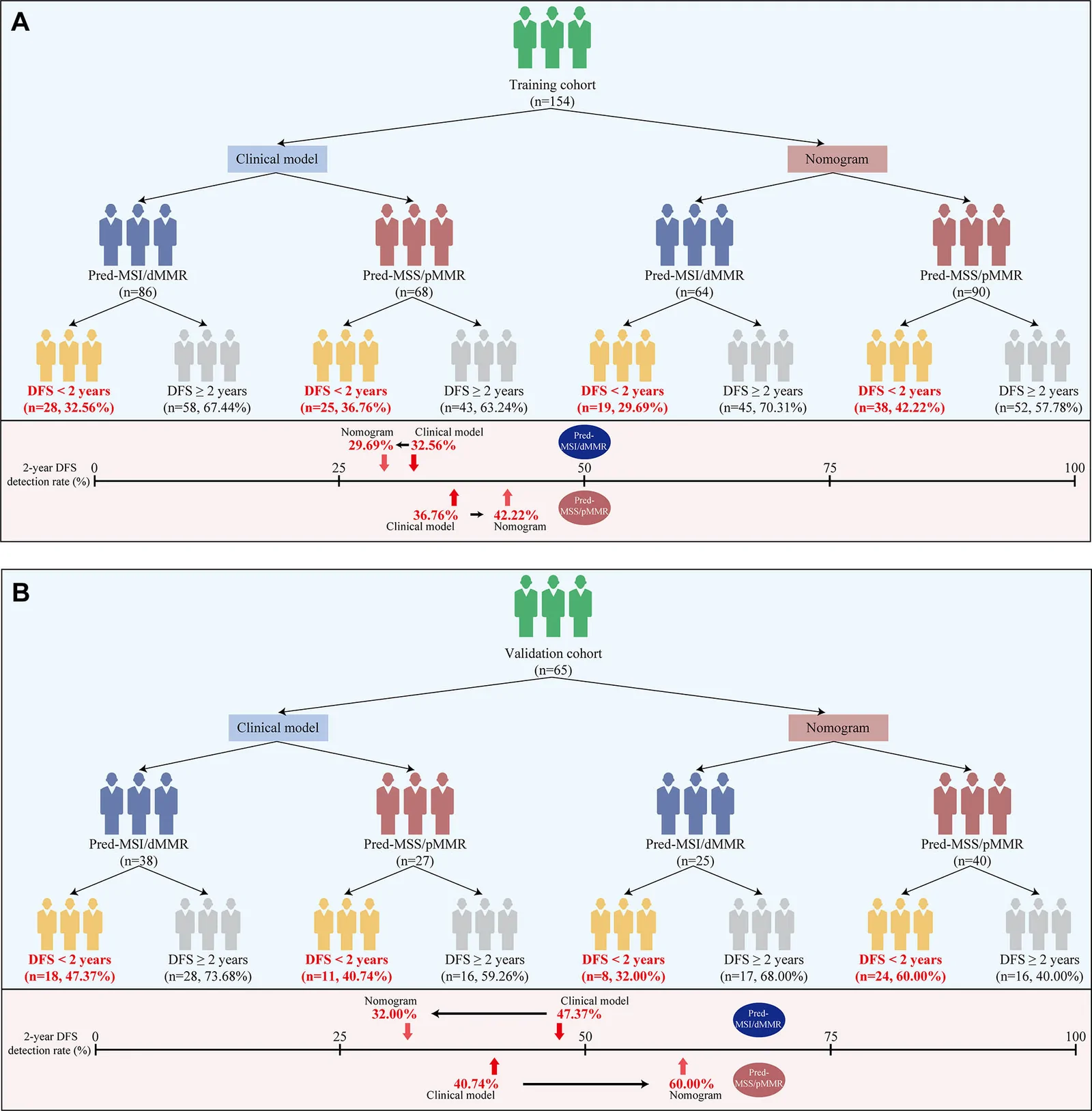
Discrimination of postoperative disease progression in patients with esophagogastric junction adenocarcinoma on the basis of the clinical model and the nomogram. The dendograms illustrate the detection rates of patients whose disease-free survival (DFS) was less than 2 years in the predicted microsatellite instability/deficient mismatch repair (Pred-MSI/dMMR) and predicted microsatellite stable/proficient mismatch repair (Pred-MSS/pMMR) groups, determined on the basis of the clinical model and the nomogram, across **(A)** the training cohort and **(B)** the validation cohort.

## Discussion

MSI/dMMR status has become a key determinant of prognosis in patients with EGJA and a critical factor in guiding personalized treatment strategies. With advances in oncologic research, current clinical guidelines strongly recommend routine preoperative assessment of this status (1). This assessment is particularly critical given that patients with MSI/dMMR status demonstrate favorable responses to immunotherapy, whereas the efficacy of conventional chemotherapy in this subgroup is limited. In this multicenter study, we developed and validated a nomogram for the preoperative prediction of MSI/dMMR status in patients with EGJA on the basis of quantitative spectral CT parameters and clinical characteristics. The results demonstrated that the AUC of the nomogram ranged from 0.86 (95% CI: 0.76, 0.96) to 0.91 (95% CI: 0.83, 0.99) across the training, validation, external test, prospective test, and neoadjuvant chemotherapy cohorts. Notably, the proposed nomogram effectively corrected misclassifications in the initial biopsies across the different cohorts. The reclassified false-negative rates ranged from 1.45% to 4.88%, and the false-positive rates ranged from 1.45% to 5.26%. Beyond its diagnostic utility, the nomogram also facilitated prognostic risk stratification for patients with EGJA in the training and validation cohorts (*P* < .001 and *P* = .003, respectively), and its score functioned as an independent predictor of DFS (*P* < .001 and *P* = .009).

Current clinical protocols and oncology guidelines generally recommend testing the MSI/dMMR status of patients with newly diagnosed EGJA based on preoperative tissue biopsy to guide precise, individualized treatment strategies (1,28). However, prior studies have indicated that both intratumoral heterogeneity and the insufficient tissue volume in biopsy samples limit the accuracy of preoperative detection of this status (16,17,29). This spatial limitation primarily stems from the fundamental nature of biopsy; endoscopic biopsy is inherently restricted to the superficial mucosal surface, providing a narrow “keyhole” view of the highly complex tumor ecosystem. Superficial biopsies may capture necrotic tissue, localized inflammatory exudates, or nonrepresentative tumor margins, thereby increasing the risk of misdiagnosis (30–32). In contrast, spectral CT enables a noninvasive, comprehensive assessment of the entire lesion within a cross-sectional section. Although the quantitative measurements in the current method are based solely on the maximum cross-sectional section, the representative plane traverses the tumor longitudinally, extending from the superficial mucosa to the deeper muscular and serosal layers. This approach comprehensively captures the heterogeneity of contrast agent uptake, mapping the varying vascular density distributions within both the dense tumor core and the invasive peripheral regions. By providing this panoramic cross-sectional perspective over the limited keyhole view of endoscopic biopsy, the constructed nomogram serves as a reliable supplementary assessment tool that mitigates the sampling bias inherent in local biopsy and effectively bridges the existing diagnostic gaps (33).

This comprehensive and complementary information offers particular value for clinical candidates receiving neoadjuvant therapy. Currently, the MSI/dMMR status of these patients is determined based on a single local biopsy, which is then used to guide the primary treatment recommendations before surgical resection. However, when faced with an erroneous or indeterminate biopsy diagnosis, patients are placed at a disadvantage because they may not receive the optimal therapeutic strategy. For example, misdiagnosed patients may undergo conventional neoadjuvant chemotherapy regimens that are toxic yet ineffective, rather than the beneficial immunotherapy specifically recommended for MSI/dMMR EGJA (14). Our study confirms that the nomogram provides an essential additional safeguard for preoperative biopsy. Although we objectively observed discrepancies between the nomogram’s predictions and the initial biopsy results, we must emphasize that this spectral CT–based approach is intended to complement the biopsy, rather than replace it. In other words, we strongly recommend that for neoadjuvant therapy candidates, initial biopsy results could be actively combined with the constructed nomogram as a dual safeguard to ensure the preoperative diagnosis of the MSI/dMMR status. Within the framework of the modern multidisciplinary team model, when discrepancies arise between the nomogram and histopathologic biopsy results, these inconsistencies could serve as critical warning signals. This alert could prompt physicians, radiologists, and pathologists to conduct a targeted reevaluation rather than blindly adhering to a single result. Furthermore, for patients whose MSI/dMMR status could not be determined via biopsy for various reasons, the outcomes predicted by this nomogram might serve as a reliable reference. In summary, the integration of the nomogram into the routine preoperative workflow establishes a reliable dual verification system, thereby improving clinical confidence.

In contrast to “black box” deep learning models that face interpretability challenges and incur high deployment costs, this nomogram is constructed using interpretable spectral parameters. Quantitative spectral CT parameters are fundamentally based on the unique attenuation characteristics of various biologic tissues and elements, thereby reflecting the close and potential associations between imaging and different pathologic statuses (34). Previous studies have shown that the CT_40keV_ attenuation and NID are directly correlated with contrast media uptake (19,20). In our study, both of these parameters were lower in patients with MSI/dMMR-type EGJA compared with patients with MSS/pMMR-type EGJA, indicating increased contrast media uptake in the MSS/pMMR group, which suggests increased blood volume, heightened vascular permeability, and an expanded interstitial space in patients with this subtype. These findings might be due to underlying biologic mechanisms of EGJA, particularly the greater angiogenesis-related gene expression and more vigorous neovascularization in patients with MSS/pMMR-type EGJA (35,36). These findings suggest that quantitative spectral CT parameters hold clinical potential for distinguishing different tissue cell statuses and microvascular landscapes within the EGJA microenvironment. The tumor microenvironment created by active neoangiogenesis and cancer-associated fibroblast proliferation is among the key factors contributing to EGJA invasiveness (37,38). This implies that tumor invasiveness could be further explored using preoperative spectral CT parameters in future research.

To our knowledge, no previous studies have comprehensively evaluated the specific utility of conventional 120-kVp CT in predicting MSI/dMMR and prognosis in patients with EGJA. In this study, we utilized the imaging characteristics of spectral CT to simulate a conventional 120-kVp–like model using 70-keV VMIs. We observed that the predictive performance of the 120-kVp–like model was lower than that of the nomogram. The performance gap might be attributed to the differences in the physical principles underlying spectral CT and conventional CT scanning. Conventional CT relies on polychromatic x-ray beams that produce averaged attenuation values, which may obscure subtle differences in tissue density due to beam-hardening artifacts and limited low-contrast resolution (39). In contrast, spectral CT leverages material decomposition algorithms and VMI technology to fundamentally enhance tissue characterization (40,41). Specifically, iodine exhibits greater attenuation at low energies because of increased photoelectric attenuation at energies approaching the iodine k edge (33.2 keV). Reconstructed VMIs at lower energies, such as 40 keV, enhance the signal-to-noise ratio of the contrast agent within microvessels (19,20). With CT_40keV_ attenuation and NID, spectral CT provides quantitative measures of microvascular alterations and subtle compositional heterogeneity in EGJA that are imperceptible on conventional CT images. Collectively, these findings underscore the potential value of spectral CT as a noninvasive adjunct to essential preoperative biopsy for achieving pathologically accurate diagnoses.

This study had several limitations. First, the inherent characteristics of EGJA, such as irregular shapes and unclear boundaries in CT images, may have led to subjective influences during manual segmentation. The inclusion of semiautomatic and automatic segmentation techniques in future studies might increase the accuracy of the nomogram. This study used consensus values from two radiologists to minimize interobserver variability for model development. Consequently, the performance of the nomogram may be inflated compared with results obtained using a single reader in actual clinical practice. Second, the prevalence of MSI/dMMR in this study exceeded previously reported values in the general population (42,43), potentially because the study population originated from high-volume specialized cancer centers. This may limit the generalizability of the findings to real-world settings. The relatively small sample size of the external test cohort limited the statistical power for subgroup analyses, which restricts the meaningful interpretation of these results. Third, the relatively short follow-up period constrained overall survival analysis. Fourth, given that the actual MSI/dMMR status affects patient prognosis directly, it is difficult to fully distinguish the prognostic value of the nomogram from the inherent survival advantages of the MSI/dMMR subtype. Therefore, the survival analysis results should be considered preliminary and exploratory.

In conclusion, we propose a noninvasive and preoperative spectral CT–based nomogram for predicting the MSI/dMMR status of EGJA, which provides a rapid assessment of EGJA prognosis. The model might serve as a valuable supplement to preoperative biopsy in clinical practice. Future efforts will focus on conducting larger-scale, multicenter cohort studies incorporating data from a broader general population to rigorously validate these findings, thereby maximizing the universal clinical applicability of the nomogram and facilitating its integration into routine precision oncology workflows.

## Supplemental Files

Appendices S1-S11, Tables S1-S11, Figures S1-S6

Conflicts of Interest
