# Supplementary material for: Spectral CT-based Nomogram for Noninvasive Assessment and Prognostic Prediction of MSI/dMMR in Esophagogastric Junction Adenocarcinoma
Source: Radiol Imaging Cancer. 2026 Sep 4;8(5):e250654. doi: 10.1148/rycan.250654 (PMC13620324; doi:10.1148/rycan.250654)
Supplement: Supplementary file 1 [file rycan250654suppa1.pdf]

Supplemental materials undergo peer review but are not copyedited, and may include typographical errors.

©RSNA, 2026
10.1148/rycan.250654

Supplementary Materials

Part I. Supplementary Information

Part II. Supplementary Tables

Part III. Supplementary Figures

Part I. Supplementary Information

Materials and methods

Appendix S1 Study design and sample

The exclusion criteria for the retrospective cohorts were as follows: (1) patients with recurrent cancer in the remnant stomach or esophagus following previous esophagogastric resections; (2) patients with other malignant tumors or other tumors at the esophagogastric junction; (3) patients with poor image quality due to severe artifacts or insufficient gastric distension; and (4) patients whose spectral CT data were missing.

The inclusion criteria for the prospective test cohort were as follows: (1) biopsy-confirmed EGJA; (2) scheduled for radical resection without neoadjuvant therapy; (3) scheduled for dual-phase contrast-enhanced spectral CT scanning of the chest, abdomen, and pelvis within 2 weeks before surgery; (4) estimated glomerular filtration rate of more than 30 mL/min/1.73 m^2; (5) scheduled for MSI/dMMR testing after resection.

The exclusion criteria for the prospective cohort were as follows: (1) patients with recurrent cancer in the remnant stomach or esophagus following previous esophagogastric resections; (2) patients with other malignant tumors or other tumors at the esophagogastric junction; (3) patients who withdrew consent to participate after enrolling; and (4) patients with poor image quality due to severe artifacts or insufficient gastric distension.

Patients did not receive preoperative neoadjuvant chemotherapy for primary patient-based (e.g., personal or family decisions to decrease treatment, financial constraints) or medical-based reasons (e.g., clinical indications requiring surgery as soon as possible [such as emergency, intestinal obstruction, or gastrointestinal bleeding], surgery-related medical policies and regulations).

Supplemental materials undergo peer review but are not copyedited, and may include typographical errors.

The inclusion criteria for the neoadjuvant chemotherapy cohort were as follows: (1) biopsy-confirmed EGJA; (2) clinically diagnosed as locally advanced stage (T3-4N+M0); (3) underwent neoadjuvant chemotherapy followed by radical resection; (4) underwent dual-phase contrast-enhanced spectral CT scanning of the chest, abdomen, and pelvis within a 2-week window surrounding the biopsy and before the neoadjuvant chemotherapy.

The exclusion criteria for the neoadjuvant chemotherapy cohort were as follows: (1) absence of MSI/dMMR status from biopsy samples; (2) absence of postoperative pathology for the assessment of Becker's tumor regression grade (TRG) staging; (3) patients with recurrent cancer in the remnant stomach or esophagus following previous esophagogastric resections; (4) patients with other malignant tumors or other tumors at the esophagogastric junction; (5) patients with poor image quality due to severe artifacts or insufficient gastric distension; and (6) patients whose spectral CT data were missing.

In accordance with current guidelines, patients received standardized postoperative treatment. The adjuvant chemotherapy regimen was determined based on pathological stage, with no distinction between patients with MSI/dMMR and MSS/pMMR statuses. Patients with pathological stage II received XELOX regimen (capecitabine combined with oxaliplatin). Patients with pathological stage III received either XELOX or SOX (S-1 plus oxaliplatin) regimen. The duration of combined chemotherapy was completed within 6 months. For patients receiving neoadjuvant chemotherapy, the protocols involved either a dual-agent regimen (combining platinum and fluoropyrimidine), or a triple-agent combination (adding a taxane to the doublet). The total duration of neoadjuvant chemotherapy did not exceed 3 months.

The sample size calculation for this study was performed using the "sample size" module in MedCalc (v.20.0.22; MedCalc Software Ltd.). For the prediction of MSI/dMMR, the minimum required sample size was calculated assuming an expected area under the curve (AUC) value of 0.80, an alpha level of 0.05, a power of 0.80, and a sample size ratio of 3.30 in the negative and positive groups (the prevalence of MSI/dMMR ranging from 13%-33%). The results showed that 9 cases were required for the MSI/dMMR group and 30 cases for the MSS/pMMR group. In this study, 154 cases were included in the training cohort (MSI/dMMR: n=35, MSS/pMMR: n=119), 65 cases in the validation cohort (MSI/dMMR: n=17, MSS/pMMR: n=48), 46 cases in the external test cohort (MSI/dMMR: n=15, MSS/pMMR: n=31), 91 cases in the prospective test cohort (MSI/dMMR: n=20, MSS/pMMR: n=71), 155 cases in the neoadjuvant chemotherapy cohort (MSI/dMMR: n=29, MSS/pMMR: n=126), respectively, meeting the requirements.

Supplemental materials undergo peer review but are not copyedited, and may include typographical errors.

For survival data(1), according to previous studies, the median disease-free survival (DFS) for the MSS/pMMR type of EGJA was assumed to be 20.70 months, and that for the MSI/dMMR type was 120 months. With a sample size ratio of 1, an alpha level of 0.05, a power of 0.80, and a 10% dropout rate, the minimum required sample size for each type was 10 cases. In the present study, the predicted MSS/pMMR group included a minimum of 40 subjects and the predicted MSI/dMMR group included at least 25 subjects, both meeting the requirements.

Appendix S2 Pathohistological evaluation

Immunohistochemical staining was performed on 3-μm-thick sections of the samples to determine the expression of four mismatch repair proteins, namely, MLH1, MSH2, MSH6, and PMS2. Normal expression of all four proteins was defined as pMMR, whereas reduced expression for any protein was defined as dMMR(2). MSI was assessed on the basis of the expression of five microsatellite markers, namely, BAT-25, BAT-26, NR-21, NR-24, and NR-27, using multiplex polymerase chain reaction or next-generation sequencing. Compared with normal tissue, tumor tissue with MSI was defined as instability in at least two markers; otherwise, it was defined as MSS(2). Within this study cohort, all patients with MSI were also confirmed as having a dMMR status, and all patients with MSS were verified as having a pMMR status. With the postoperative pathological results as the reference standard, the patients in the training, validation, external, and prospective test cohorts were categorized into MSI/dMMR and MSS/pMMR groups. In contrast, those in the neoadjuvant chemotherapy cohort were grouped according to the preoperative biopsy results. These classifications served as the reference standard for evaluating model performance. In the neoadjuvant therapy cohort, the pathological response of surgical samples was evaluated according to Becker's tumor regression grade (TRG; 1a, 1b, 2, 3)(3). A major pathological response (MPR) was defined as <10% viable tumor cells (reclassified from Becker grades 1a+1b). Patients with TRG 2 or 3 (reflecting ≥10% viable tumor cells) were categorized as non-MPR.

Appendix S3 Clinicopathological data collection

Patients with early-stage EGJA were seen every 6 months for the first two years and annually thereafter. For patients with advanced-stage EGJA, the frequency was every 3 months for the first two years, followed by every 6 months thereafter. Patients with Siewert types I and II were staged according to esophageal carcinoma, and those with Siewert type III were staged according to gastric carcinoma. Early-

Supplemental materials undergo peer review but are not copyedited, and may include typographical errors.

stage EGJA was defined as tumor invasion confined to the mucosa or submucosa (T1 stage), irrespective of regional lymph node involvement. Advanced-stage EGJA was defined as lesions that had penetrated beyond the submucosa (T2-4 stage), irrespective of regional lymph node involvement. Data were obtained through telephone interviews or by reviewing clinical records. If the patients could not be contacted during the scheduled telephone interview (unreachable on 3 days within one month), they were considered lost to follow-up. Postoperative follow-up was systematically conducted through clinical evaluations, contrast-enhanced CT and endoscopic biopsies. Local progression or recurrence was confirmed based on radiological findings and/or definitive histopathological evidence(4). Disease-free survival (DFS) time was defined as the time interval between the date of surgery and the first date of disease progression or the last follow-up date (May 2025), whichever occurred earlier. Disease progression refers to local recurrence, distant metastasis detected by imaging or pathology, or death.

Appendix S4 Collection and postprocessing of spectral CT data

Patients fasted for 8 hours prior to the scan. Fifteen minutes before the scan, the patients consumed 800–1000 mL of water to fully fill the gastric cavity and the small bowel lumen. Patients were examined in the craniocaudal direction and a supine position during breath-holding. The CT scan covered the chest, abdomen and pelvis, including the nonenhanced phase, arterial phase (AP), and venous phase (VP). For enhanced CT scanning, a nonionic contrast agent (Ioversol Injection 320 mgI/mL; Jiangsu Hengrui Pharmaceutical Co., Ltd.) was delivered at a standard dose (1.5 mL/kg) via the elbow vein by a pump injector (EmpowerCTA+, Bracco Injenerring S.A.) at a rate of 2.5–3 mL/s, followed by 30 mL of saline at the same rate. AP and VP scanning were performed 35–38 s and 90 s after the initiation of contrast agent injection, respectively. The procedure was performed in accordance with international practices and standards(5, 6). Other acquisition parameters were as follows: (1) automatic exposure control using a 3D DoseRight Index (Philips Healthcare) with exposures of 250–300 mAs, (2) detector collimation of 64×0.625 mm, (3) a tube voltage of 120 kVp, (4) a helical pitch of 1.172, (5) a rotation time of 0.75 s, and (6) an image matrix of 512×512.

The spectral-based images were reconstructed using a spectral reconstruction algorithm (Spectral, level 3, Kernel B, Philips Healthcare). The slice thickness of all axial images was 1.0 mm, with a section increment of 1.0 mm. The reconstructed spectral images included virtual monoenergetic images (VMIs), effective atomic number (Z_{eff}) maps, and iodine density (ID) maps.

Supplemental materials undergo peer review but are not copyedited, and may include typographical errors.

Appendix S5 Image analysis and parameter measurement

The T stage reflects the depth of tumor invasion, which is assessed by the degree of involvement of the esophageal or gastric wall layers (T1: tumor invades the lamina propria, muscularis mucosae, or submucosa; T2: tumor invades the muscularis propria; T3: gastric tumor invades the subserosal connective tissue without invasion of the visceral peritoneum or adjacent structures or esophageal tumor invades the adventitia; T4: gastric tumor invades the serosa (visceral peritoneum) or adjacent structures or esophageal tumor invades adjacent structures). The N stage reflects the regional lymph node involvement (N0: no regional lymph node metastasis; N1-3: regional lymph node metastasis)(4).With respect to the VMIs (from 40 keV to 100 keV, with intervals of 10 keV), Z_{eff}, and ID maps, each observer manually contoured the tumor as the region of interest (ROI) in the slice with the longest axial tumor diameter, as well as in the upper and lower slices (ROIs ranged from 0.59 cm^2 to 22.38 cm^2), and the CT_{tumor}, Z_{eff_tumor} and ID_{tumor} values were recorded. The ROIs were manually delineated within the descending aorta lumen in the same slice (ROIs ranged from 1.72 cm^2 to 2.45 cm^2), and the CT_{aorta}, Z_{eff_aorta} and ID_{aorta} values were recorded. The final results were averaged across the three ROIs. All ROI markings for the same patient were automatically copied onto the VMI, Z_{eff}, and ID maps for the corresponding phase to generate the corresponding parameter values. The following quantitative spectral CT parameters were calculated: (1) the normalized effective atomic number (NZ_{eff}): $NZ_{eff}=Z_{eff\_tumor}/Z_{eff\_aorta}$, and (2) the normalized iodine density (NID): $NID=ID_{tumor}/ID_{aorta}$.

The Z_{eff} value reflects tissue characteristics based on atomic composition, and the ID value represents the lesion's degree of vascularization based on iodine concentration(7, 8). Normalized parameters relative to the descending aorta minimize interpatient variability caused by differences in cardiac output, contrast agent injection protocol, and acquisition time, thereby achieving more accurate comparisons(7). Lower NZ_{eff} and NID values are associated with MSI/dMMR status due to changes in the tumor microenvironment(9).

Appendix S6 Model development

Following the aforementioned parameter measurement methods, we recorded CT_{70keV_tumor} and CT_{70keV_aorta}. The normalized tumor CT attenuation value (NCT_{70keV_tumor}) was calculated as $CT_{70keV\_tumor}/CT_{70keV\_aorta}$. We then constructed four 120 kVp-like models: (1) CT_{70keV_tumor} model; (2)

Supplemental materials undergo peer review but are not copyedited, and may include typographical errors.

NCT_{70keV_tumor} model; (3) a combined model incorporating CT_{70keV_tumor} and NCT_{70keV_tumor}; and (4) a combined model integrating CT_{70keV_tumor}, NCT_{70keV_tumor}, and the selected clinical predictors identified in the multivariable analysis.

Appendix S7 Statistical analysis

Student's *t* test or the Mann–Whitney *U* test was used to compare continuous variables. Categorical variables were analyzed with the chi-square test or Fisher's exact test, as appropriate. Interobserver agreement was assessed using the intraclass correlation coefficient (ICC) and Cohen's kappa coefficient (κ). Since mismatch repair data were more comprehensively available from biopsies than from MSI testing results, a concordance analysis for biopsy was performed using mismatch repair status. The κ values were utilized to assess the diagnostic concordance across pathological classifications of biopsy samples, spectral CT and nomogram model predictions, and surgical resection samples. We also constructed pairwise confusion matrices for these three outcomes.

The DeLong's test was used to compare the AUC values across different models, and the difference between AUC values (ΔAUC) was calculated. The accuracy, sensitivity, specificity, positive predictive value, and negative predictive value were calculated. The differences in diagnostic accuracy, sensitivity, and specificity were assessed using McNemar's test. The Akaike information criterion (AIC) was used to evaluate and compare the goodness-of-fit among different models, with a lower AIC value indicating a superior model. Univariable and multivariable Cox analyses were conducted to identify independent factors for DFS, and the hazard ratios (HRs) with 95% CIs were reported. First, univariable Cox proportional hazards regression analyses were used to assess the association between each variable and survival outcomes. Variables that showed statistical significance in the univariable analysis were subsequently included in the multivariable Cox regression analysis. Prior to multivariable logistic regression analysis, collinearity diagnostics were performed to assess potential multicollinearity among the candidate predictors. An auxiliary linear regression was conducted to regress each predictor against all remaining predictors to calculate the variance inflation factors (VIFs). Predictors with a VIF < 3 were considered to have no significant multicollinearity and were subsequently included in the final multivariable analysis. To prevent multicollinearity and overfitting, a backward stepwise selection method based on the AIC was then employed to construct the optimal multivariable model. The proportional hazards assumption was verified using the Schoenfeld residuals test. Bonferroni correction

Supplemental materials undergo peer review but are not copyedited, and may include typographical errors.

was used for adjusted *P* values involving multiple comparisons.

Results

Appendix S8 Clinicopathological characteristics

This study enrolled EGJA patients prospectively and retrospectively from Center 1 (the First Affiliated Hospital of Fujian Medical University) and retrospectively from Center 2 (Zhongshan Hospital Xiamen University).

Between May 2021 and April 2024, 305 patients with EGJA meeting the inclusion criteria at Center 1 were retrospectively enrolled in the study, among whom 86 were excluded. Patients were excluded for recurrent cancer in the remnant stomach or esophagus following previous esophagogastric resections (n=14), other malignant tumors or other tumors at the esophagogastric junction (n=36), poor image quality (n=25), and missing spectral CT data (n=11). Ultimately, 219 patients were included and randomly divided into a training cohort (n=154) and a validation cohort (n=65) in a ratio of 7:3.

From August 2023 to March 2025, 60 patients with EGJA eligible for inclusion at Center 2 were retrospectively recruited into the study. Among them, 14 patients were excluded. Patients were excluded due to recurrent cancer in the remnant stomach or esophagus following previous esophagogastric resections (n=1), other malignant tumors or other tumors at the esophagogastric junction (n=6), poor image quality (n=5), and missing spectral CT data (n=2). Finally, 46 patients were included in the external test cohort.

During April 2025 through January 2026, prospective enrollment of 111 participants with EGJA at Center 1 was conducted, of whom 20 were excluded. Participants were excluded as follows: those with recurrent cancer in the remnant stomach or esophagus following previous esophagogastric resections (n=6), those with other malignant tumors or other tumors at the esophagogastric junction (n=12), patients who withdrew consent to participate after enrolling (n=0), and those with poor image quality (n=2). A total of 91 participants were ultimately included in the prospective test cohort.

In the period from May 2021 to December 2025, 203 patients with EGJA receiving neoadjuvant chemotherapy from Center 1 and Center 2 were enrolled. Among them, 48 patients were excluded, leaving a final cohort of 155 patients in the neoadjuvant chemotherapy cohort. Patients were excluded due to absence of MSI/dMMR status from biopsy samples (n=26), absence of postoperative pathology for TRG grade (n=4), recurrent cancer in the remnant stomach or esophagus following previous

Supplemental materials undergo peer review but are not copyedited, and may include typographical errors.

esophagogastric resections (n=0), other malignant tumors or other tumors at the esophagogastric junction (n=3), or poor image quality (n=8), and missing spectral CT data (n=7).

Appendix S9 Spectral CT model performance

The AUC of the spectral CT model was higher than that of the clinical model in the training cohort (AUC, 0.81 [95% CI: 0.72, 0.89] vs 0.75 [95% CI: 0.66, 0.84], respectively; *P* = .42), the validation cohort (AUC, 0.83 [95% CI: 0.71, 0.95] vs 0.72 [95% CI: 0.59, 0.85], respectively; *P* = .22), the external test cohort (AUC, 0.81 [95% CI: 0.68, 0.95] vs 0.72 [95% CI: 0.56, 0.88], respectively; *P* = .45), the prospective test cohort (AUC, 0.84 [95% CI: 0.75, 0.94] vs 0.69 [95% CI: 0.57, 0.81], respectively; *P* = .08), and the neoadjuvant chemotherapy cohort (AUC, 0.81 [95% CI: 0.70, 0.92] vs 0.72 [95% CI: 0.62, 0.81], respectively; *P* = .28) (Table S7).

Appendix S10 Nomogram performance

Compared with the clinical model, the nomogram demonstrated higher efficacy for predicting MSI/dMMR across all cohorts, including the training cohort (AUC, 0.87 [95% CI: 0.81, 0.93] vs 0.75 [95% CI: 0.66, 0.84], respectively; *P* = .002), the validation cohort (AUC, 0.87 [95% CI: 0.78, 0.96] vs 0.72 [95% CI: 0.59, 0.85], respectively; *P* = .003), the external test cohort (AUC, 0.91 [95% CI: 0.83, 0.99] vs 0.72 [95% CI: 0.56, 0.88], respectively; *P* = .009), the prospective test cohort (AUC, 0.89 [95% CI: 0.80, 0.98] vs 0.69 [95% CI: 0.57, 0.81], respectively; *P* < .001), and the neoadjuvant chemotherapy cohort (AUC, 0.86 [95% CI: 0.76, 0.96] vs 0.72 [95% CI: 0.62, 0.81], respectively; *P* = .02) (Table S7).

Furthermore, the sensitivity of the nomogram was better than that of the spectral CT model in the training cohort (Sensitivity, 89% [95% CI: 73, 97] vs 71% [95% CI: 54, 85], respectively; *P* = .08), the validation cohort (Sensitivity, 94% [95% CI: 71, 100] vs 71% [95% CI: 44, 90], respectively; *P* = .13), the external test cohort (Sensitivity, 100% [95% CI: 78, 100] vs 93% [95% CI: 68, 100], respectively; *P* > .99), and the neoadjuvant chemotherapy cohort (Sensitivity, 76% [95% CI: 56, 90] vs 59% [95% CI: 39, 76], respectively; *P* = .18).

The nomogram demonstrated robust predictive performance, with 92% (120/130) of the subgroups showing AUCs exceeding 0.80 (Fig. S2). Specifically, within these subgroups, AUCs ranged from 0.81 (95% CI: 0.73, 0.88; cN0 subgroup of all cohorts) to 0.98 (95% CI: 0.94, 1.00; Siewert type II subgroup of the prospective test cohort). Although minor fluctuations (AUC = 1.00 or < 0.70) occurred in 8 extreme

Supplemental materials undergo peer review but are not copyedited, and may include typographical errors.

subgroups, this was primarily owing to limited sample sizes (n < 10) or substantial variations between patients with MSI/dMMR and MSS/pMMR. For example, only 1 subgroup had an AUC of 0.66 (95% CI: 0.00, 1.00; Siewert type I subgroup of the neoadjuvant chemotherapy cohort, 4 vs 38 patients). An AUC of 1.00 (95% CI: 1.00, 1.00) was observed in 7 subgroups, including the validation cohort (well-differentiated subgroup: 1 vs 8 patients), the external test cohort (elevated CEA subgroup: 3 vs 6 patients; moderately differentiated subgroup: 2 vs 7 patients; cT1-2 stage subgroup: 5 vs 6 patients), the prospective test cohort (well-differentiated subgroup: 3 vs 11 patients; intestinal type subgroup: 4 vs 24 patients), and the neoadjuvant chemotherapy cohort (elevated CA199 subgroup: 5 vs 27 patients). In summary, the overall consistency of the nomogram across diverse clinical stratifications confirmed its reliable utility and robustness.

Appendix S11 Nomogram performance for prognosis

Kaplan–Meier survival curves indicated that the nomogram risk-stratified patients with EGJA (Fig 6). In the training cohort, median DFS based on the reference standard was 29.10 months for patients with MSS/pMMR and 42.40 months for patients with MSI/dMMR (Fig 6a). The median DFS predicted by the nomogram was 27.70 months for patients with MSS/pMMR and 36.20 months for patients with MSI/dMMR (Fig 6a). This stratification capability was further confirmed in the validation cohort. The median DFS for the MSS/pMMR group based on the reference standard classification was 28.70 months, and 39.60 months for the MSI/dMMR group (Fig 6b). The nomogram predictions accurately reproduced the same median DFS (28.70 months vs 39.60 months) (Fig 6b).

Supplemental materials undergo peer review but are not copyedited, and may include typographical errors.

References

1. Smyth EC, Wotherspoon A, Peckitt C, Gonzalez D, Hulkki-Wilson S, Eltahir Z, Fassan M, Rugge M, Valeri N, Okines A, Hewish M, Allum W, Stenning S, Nankivell M, Langley R, Cunningham D. Mismatch Repair Deficiency, Microsatellite Instability, and Survival: An Exploratory Analysis of the Medical Research Council Adjuvant Gastric Infusional Chemotherapy (MAGIC) Trial. JAMA Oncology 2017;3(9):1197-1203. doi: 10.1001/jamaoncol.2016.6762

2. Luchini C, Bibeau F, Ligtenberg MJL, Singh N, Nottegar A, Bosse T, Miller R, Riaz N, Douillard JY, Andre F, Scarpa A. ESMO recommendations on microsatellite instability testing for immunotherapy in cancer, and its relationship with PD-1/PD-L1 expression and tumour mutational burden: a systematic review-based approach. Annals of Oncology 2019;30(8):1232-1243. doi: 10.1093/annonc/mdz116

3. Becker K, Mueller JD, Schulmacher C, Ott K, Fink U, Busch R, Böttcher K, Siewert JR, Höfler H. Histomorphology and grading of regression in gastric carcinoma treated with neoadjuvant chemotherapy. Cancer 2003;98(7):1521-1530. doi: 10.1002/cncr.11660

4. Ajani JA, D'Amico TA, Bentrem DJ, Chakrabarti S, Corvera C, Das P, Enzinger PC, Enzler T. Esophageal and Esophagogastric Junction Cancers, Version 2.2026. National Comprehensive Cancer Network;January 21, 2026.:https://www.nccn.org/professionals/physician_gls/pdf/esophageal.pdf.

5. Zhengyu J. China expert consensus on clinical application of dual-layer spectral detector CT. Chin J Radiol 2020;54(7):635-643. doi: 10.3760/cma.j.cn112149-20200513-00679

6. Karout L, Kalra MK. Survey of CT radiation doses and iodinated contrast medium administration: an international multicentric study. Eur Radiol 2025;35(4):1915-1932. doi: 10.1007/s00330-024-11017-7

7. Garcia-Figueiras R, Baleato-Gonzalez S, Busquets-Carrera O, Fraga-Pineiro C, Luna A. Quantitative Dual-Energy CT in Abdominal Imaging: Technical Considerations and Emerging Clinical Applications. Radiographics 2026;46(3):e250074. doi: 10.1148/rg.250074

8. Rassouli N, Etesami M, Dhanantwari A, Rajiah P. Detector-based spectral CT with a novel dual-layer technology: principles and applications. Insights into Imaging 2017;8(6):589-598. doi: 10.1007/s13244-017-0571-4

9. Zhu Y, Wang P, Wang B, Jiang Z, Li Y, Jiang J, Zhong Y, Xue L, Jiang L. Dual-layer spectral-detector CT for predicting microsatellite instability status and prognosis in locally advanced gastric cancer. Insights into Imaging 2023;14(1). doi: 10.1186/s13244-023-01490-x

Supplemental materials undergo peer review but are not copyedited, and may include typographical errors.

Part II. Supplementary Tables

Table S1 Clinicopathological Variables of Patients among Different Cohorts

Variables	Training cohort (n=154)		Validation cohort (n=65)		External test cohort (n=46)		Prospective test cohort (n=91)		Neoadjuvant chemotherapy cohort (n=155)	
	MSI/dMMR (n=35)	MSS/pMMR (n=119)	MSI/dMMR (n=17)	MSS/pMMR (n=48)	MSI/dMMR (n=15)	MSS/pMMR (n=31)	MSI/dMMR (n=20)	MSS/pMMR (n=71)	MSI/dMMR (n=29)	MSS/pMMR (n=126)
Clinicopathological variables										
Siewert type										
I	11 (31)	44 (37)	3 (18)	13 (27)	4 (27)	8 (26)	5 (25)	22 (31)	4 (14)	38 (30)
II	7 (20)	41 (34)	7 (41)	16 (33)	3 (20)	7 (23)	7 (35)	27 (38)	12 (41)	40 (32)
III	17 (49)	34 (29)	7 (41)	19 (40)	8 (53)	16 (51)	8 (40)	22 (31)	13 (45)	48 (38)
Lauren type										
Intestinal type	18 (52)	43 (36)	8 (47)	20 (42)	6 (40)	10 (32)	4 (20)	24 (34)	8 (28)	26 (21)
Mixed type	5 (14)	43 (36)	4 (24)	18 (37)	4 (27)	9 (29)	9 (45)	28 (39)	9 (31)	16 (13)
Diffuse type	12 (34)	33 (28)	5 (29)	10 (21)	5 (33)	12 (39)	7 (35)	19 (27)	9 (31)	24 (19)
NA	0 (0)	0 (0)	0 (0)	0 (0)	0 (0)	0 (0)	0 (0)	0 (0)	3 (10)	60 (47)
Histological grade										
Well	8 (23)	12 (10)	1 (6)	8 (17)	6 (40)	8 (26)	3 (15)	11 (15)	0 (0)	8 (6)
Moderate	13 (37)	52 (44)	8 (47)	18 (37)	2 (13)	7 (23)	7 (35)	22 (31)	6 (21)	21 (17)
Poor	14 (40)	55 (46)	8 (47)	22 (46)	7 (47)	16 (51)	10 (50)	38 (54)	20 (69)	37 (29)

Supplemental materials undergo peer review but are not copyedited, and may include typographical errors.

NA	0 (0)	0 (0)	0 (0)	0 (0)	0 (0)	0 (0)	0 (0)	0 (0)	3 (10)	60 (48)
Pathological response										
MPR	NA	NA	NA	NA	NA	NA	NA	NA	4 (14)	64 (51)
Non-MPR	NA	NA	NA	NA	NA	NA	NA	NA	25 (86)	62 (49)
Spectral CT parameters										
Maximum diameter	5.10 (3.20 – 6.40)	4.20 (2.80 – 5.70)	4.30 (3.35 – 5.50)	4.10 (2.78 – 6.20)	3.50 (1.50 – 4.50)	3.90 (2.80 – 6.50)	4.30 (2.03 – 6.73)	3.30 (1.90 – 4.20)	4.80 (3.35 – 5.75)	4.85 (3.30 – 6.00)
Arterial phase										
CT_{40keV}	214.70 (176.10 – 255.20)	237.00 (213.80 – 261.80)	221.70 (208.95 – 261.65)	242.80 (224.63 – 278.58)	233.90 (193.50 – 251.20)	251.30 (216.30 – 271.00)	221.90 (200.15 – 262.45)	244.30 (213.70 – 268.00)	232.30 (197.00 – 242.75)	245.70 (234.38 – 251.60)
CT_{50keV}	148.20 (127.20 – 170.40)	163.70 (140.20 – 181.60)	156.10 (138.85 – 176.35)	176.90 (152.90 – 192.55)	171.90 (151.50 – 214.10)	175.30 (138.70 – 185.30)	159.60 (147.85 – 167.70)	173.30 (146.20 – 189.80)	157.40 (150.15 – 169.10)	166.95 (159.78 – 174.10)
CT_{60keV}	115.70 (103.10 – 135.00)	119.30 (107.80 – 130.70)	116.10 (110.35 – 132.30)	121.95 (114.15 – 135.08)	116.80 (106.00 – 135.90)	129.10 (106.20 – 132.70)	116.55 (110.08 – 124.50)	119.10 (109.40 – 129.90)	116.80 (108.95 – 123.00)	122.90 (117.00 – 127.88)
CT_{70keV}	92.90 (84.10 – 107.50)	94.50 (86.60 – 104.20)	94.70 (92.45 – 104.65)	97.95 (89.50 – 109.85)	92.60 (84.70 – 101.90)	95.30 (88.30 – 110.00)	95.05 (91.78 – 100.95)	95.20 (88.80 – 102.50)	95.60 (90.50 – 101.15)	95.90 (90.23 – 102.43)
CT_{80keV}	80.00 (74.40 – 86.30)	82.50 (75.60 – 88.35)	86.20 (78.50 – 88.60)	83.55 (78.23 – 90.63)	83.40 (71.60 – 88.00)	80.70 (74.30 – 96.50)	80.70 (75.63 – 91.05)	80.40 (75.60 – 85.20)	83.90 (81.90 – 87.30)	81.30 (75.28 – 85.40)

Supplemental materials undergo peer review but are not copyedited, and may include typographical errors.

CT_{90keV}	72.40 (66.00 – 79.20)	73.95 (68.70 – 78.50)	78.90 (67.50 – 81.50)	73.80 (70.18 – 79.53)	72.20 (63.40 – 79.70)	73.10 (65.10 – 82.30)	72.20 (69.10 – 79.10)	73.60 (68.40 – 77.50)	75.40 (69.50 – 80.50)	72.90 (69.48 – 75.85)
CT_{100keV}	66.30 (60.40 – 71.70)	66.20 (62.40 – 71.80)	71.60 (61.25 – 77.95)	66.30 (63.25 – 71.90)	66.00 (57.70 – 71.20)	66.00 (62.40 – 76.20)	67.25 (60.58 – 71.23)	67.90 (61.50 – 72.50)	68.30 (65.95 – 71.90)	68.60 (65.95 – 70.73)
ID	1.92 (1.43 – 2.36)	2.21 (1.86 – 2.75)	2.03 (1.51 – 2.83)	2.10 (1.67 – 2.44)	2.06 (1.79 – 2.51)	2.35 (1.86 – 2.81)	2.19 (1.83 – 2.57)	2.22 (1.74 – 2.53)	2.10 (1.82 – 2.38)	2.21 (1.83 – 2.63)
NID	0.20 (0.15 – 0.27)	0.24 (0.19 – 0.30)	0.23 (0.14 – 0.31)	0.24 (0.20 – 0.29)	0.25 (0.21 – 0.34)	0.25 (0.21 – 0.31)	0.23 (0.17 – 0.28)	0.23 (0.19 – 0.29)	0.19 (0.15 – 0.30)	0.23 (0.18 – 0.29)
Z_{eff}	8.23 (7.97 – 8.51)	8.35 (8.08 – 8.54)	8.38 (8.25 – 8.72)	8.45 (8.12 – 8.65)	8.34 (8.03 – 8.64)	8.36 (7.97 – 8.51)	8.34 (7.95 – 8.71)	8.41 (8.06 – 8.59)	8.47 (8.07 – 8.70)	8.31 (8.08 – 8.59)
NZ_{eff}	0.78 (0.73 – 0.84)	0.80 (0.76 – 0.87)	0.80 (0.76 – 0.87)	0.82 (0.76 – 0.85)	0.77 (0.73 – 0.84)	0.83 (0.75 – 0.88)	0.79 (0.74 – 0.86)	0.83 (0.77 – 0.87)	0.82 (0.79 – 0.85)	0.82 (0.75 – 0.88)
Venous phase										
CT_{40keV}	224.80 (212.20 – 265.80)	272.90 (242.40 – 295.60)	236.40 (214.50 – 265.25)	261.50 (247.65 – 300.08)	228.20 (203.20 – 261.40)	250.90 (217.60 – 275.90)	244.60 (220.75 – 271.23)	280.10 (264.80 – 301.40)	247.50 (211.20 – 267.30)	263.00 (246.23 – 291.40)
CT_{50keV}	169.65 (155.30 – 185.70)	180.60 (152.75 – 203.90)	169.20 (162.80 – 178.15)	178.85 (169.75 – 208.85)	164.10 (155.70 – 185.60)	178.20 (147.80 – 203.90)	176.05 (128.00 – 215.23)	181.30 (157.20 – 196.30)	173.50 (125.30 – 212.20)	179.55 (160.20 – 191.78)
CT_{60keV}	128.20 (116.85 – 142.90)	132.15 (114.85 – 148.90)	126.30 (123.40 – 135.70)	138.75 (127.70 – 149.95)	123.70 (113.80 – 145.50)	127.40 (112.90 – 145.30)	113.05 (104.78 – 149.13)	131.80 (120.70 – 141.90)	111.60 (102.95 – 141.00)	131.15 (120.38 – 144.33)

Supplemental materials undergo peer review but are not copyedited, and may include typographical errors.

CT_{70keV}	101.70 (91.80 – 111.70)	105.20 (87.20 – 114.60)	98.40 (95.60 – 105.10)	111.15 (99.28 – 120.65)	95.70 (84.30 – 103.60)	103.50 (88.40 – 119.10)	95.60 (87.50 – 103.23)	102.20 (96.20 – 109.40)	94.40 (85.15 – 114.65)	103.40 (95.88 – 110.45)
CT_{80ke})	85.20 (78.60 – 92.70)	85.60 (76.60 – 95.20)	83.30 (77.90 – 93.25)	92.05 (80.73 – 102.18)	81.90 (73.20 – 86.20)	81.40 (73.40 – 101.80)	81.90 (77.13 – 89.45)	85.30 (80.80 – 89.50)	83.20 (75.25 – 94.30)	85.95 (80.83 – 91.70)
CT_{90keV}	76.50 (70.65 – 83.40)	76.80 (67.60 – 84.60)	73.90 (71.95 – 79.35)	78.55 (72.38 – 86.45)	70.60 (68.90 – 72.60)	73.90 (62.70 – 85.90)	72.40 (68.58 – 79.85)	76.80 (71.40 – 80.00)	73.90 (67.60 – 83.05)	76.70 (70.88 – 82.20)
CT_{100keV}	70.50 (63.30 – 74.50)	68.10 (58.80 – 76.30)	68.60 (62.65 – 73.20)	69.95 (64.33 – 76.35)	65.10 (62.10 – 66.70)	66.90 (57.00 – 78.00)	68.45 (62.90 – 75.90)	69.10 (64.40 – 73.60)	66.00 (61.60 – 75.15)	70.20 (64.78 – 75.60)
ID	2.32 (1.86 – 2.90)	2.40 (1.99 – 2.83)	2.48 (2.12 – 3.10)	2.40 (2.16 – 2.90)	2.28 (2.12 – 2.48)	2.38 (1.70 – 2.85)	2.21 (1.74 – 2.78)	2.48 (2.00 – 2.76)	2.58 (2.01 – 2.86)	2.47 (2.17 – 2.77)
NID	0.55 (0.46 – 0.63)	0.67 (0.59 – 0.72)	0.54 (0.50 – 0.66)	0.70 (0.63 – 0.76)	0.55 (0.51 – 0.59)	0.71 (0.58 – 0.75)	0.63 (0.60 – 0.67)	0.70 (0.64 – 0.78)	0.62 (0.51 – 0.65)	0.68 (0.64 – 0.73)
Z_{eff}	8.44 (8.19 – 8.71)	8.53 (8.24 – 8.57)	8.47 (8.23 – 8.67)	8.51 (8.20 – 8.67)	8.45 (8.09 – 8.64)	8.51 (8.39 – 8.69)	8.51 (8.33 – 8.66)	8.42 (8.23 – 8.59)	8.53 (8.22 – 8.72)	8.48 (8.25 – 8.66)
NZ_{eff}	0.89 (0.85 – 0.91)	0.91 (0.87 – 0.93)	0.86 (0.85 – 0.89)	0.92 (0.88 – 0.93)	0.85 (0.83 – 0.88)	0.91 (0.88 – 0.93)	0.87 (0.82 – 0.89)	0.88 (0.83 – 0.92)	0.86 (0.80 – 0.88)	0.89 (0.84 – 0.92)

NOTE. — For Clinicopathological variable, data are reported as numbers of patients with percentages in parentheses. For Spectral CT parameters, data are reported as medians with IQRs in parentheses. For the neoadjuvant chemotherapy cohort, the classification into MSI/dMMR and MSS/pMMR was based on the pathological results from preoperative biopsy specimens. Maximum diameter was the longest tumor diameter measured on the axial plane in the 40 keV VMI during the VP. MPR = major pathological

Supplemental materials undergo peer review but are not copyedited, and may include typographical errors.

response; ID = iodine density; NID = normalized iodine density; Z_{eff} = effective atomic number; NZ_{eff} = normalized effective atomic number; MSI/dMMR = Microsatellite instability/ Deficient mismatch repair; MSS/pMMR = Microsatellite stable/ Proficient mismatch repair; NA = not applicable.

Supplemental materials undergo peer review but are not copyedited, and may include typographical errors.

Table S2 Interobserver Agreement for Quantitative Data

Quantitative data	arterial phase			venous phase		
	Observer 1	Observer 2	ICC value	Observer 1	Observer 2	ICC value
Maximum diameter				4.00 (2.30, 5.70)	4.10 (2.40, 5.80)	0.85 (0.82, 0.87)
CT_{40keV}	216.20 (192.10, 242.40)	217.80 (191.50, 245.20)	0.90 (0.88, 0.91)	251.80 (214.80, 283.00)	250.10 (217.30, 283.30)	0.85 (0.83, 0.87)
CT_{50keV}	148.30 (127.10, 175.30)	153.20 (129.60, 179.30)	0.89 (0.87, 0.91)	183.40 (165.10, 202.30)	182.90 (162.40, 202.80)	0.86 (0.83, 0.88)
CT_{60keV}	117.30 (103.60, 130.50)	117.80 (104.50, 133.70)	0.88 (0.86, 0.90)	136.30 (119.50, 152.10)	137.50 (119.10, 156.40)	0.90 (0.88, 0.92)
CT_{70keV}	92.80 (79.20, 105.60)	91.90 (78.10, 106.30)	0.90 (0.88, 0.91)	108.50 (96.20, 122.80)	108.80 (91.30, 124.50)	0.88 (0.86, 0.90)
CT_{80keV}	79.20 (70.00, 88.10)	79.20 (69.70, 88.40)	0.91 (0.89, 0.92)	95.50 (86.60, 104.80)	94.70 (84.10, 106.10)	0.91 (0.89, 0.92)
CT_{90keV}	70.00 (62.50, 78.20)	70.70 (62.40, 77.70)	0.89 (0.87, 0.91)	86.00 (77.00, 94.10)	85.20 (74.70, 94.20)	0.89 (0.87, 0.91)
CT_{100keV}	65.80 (60.10, 72.40)	66.10 (59.80, 71.80)	0.89 (0.88, 0.91)	78.70 (71.00, 84.70)	77.30 (68.50, 84.60)	0.91 (0.89, 0.93)
ID_{tumor}	2.13 (1.74, 2.50)	2.06 (1.65, 2.45)	0.92 (0.90, 0.93)	2.30 (1.91, 2.81)	2.35 (1.91, 2.79)	0.93 (0.92, 0.94)
ID_{aorta}	9.54	9.48	0.91	3.79	3.73	0.96

Supplemental materials undergo peer review but are not copyedited, and may include typographical errors.

	(7.64, 10.43)	(7.72, 10.36)	(0.89, 0.93)	(3.27, 4.48)	(3.21, 4.43)	(0.95, 0.97)
Z_{eff_tumor}	8.34	8.34	0.92	8.48	8.44	0.90
	(8.06, 8.58)	(8.07, 8.57)	(0.90, 0.93)	(8.18, 8.76)	(8.15, 8.68)	(0.88, 0.92)
Z_{eff_aorta}	10.23	10.26	0.94	9.07	9.10	0.91
	(9.23, 11.05)	(9.27, 11.01)	(0.93, 0.95)	(8.87, 9.32)	(8.89, 9.43)	(0.89, 0.92)

NOTE. — For observer 1 and observer 2, data are reported as medians with IQRs in parentheses. For intraclass correlation coefficients, data in parentheses are 95% CI. Maximum diameter was the longest tumor diameter measured on the axial plane in the 40 keV VMI during the VP. ICC = intraclass correlation coefficients; ID = iodine density; Z_{eff} = effective atomic number.

Supplemental materials undergo peer review but are not copyedited, and may include typographical errors.

Table S3 Interobserver Agreement for Subjective Imaging Features

Subjective features	Observer 1	Observer 2	Disagreement	Final decision	Kappa value
cT stage					0.84 (0.80, 0.88)
T1-2 (%)	18 (92/511)	22 (113/511)	4 (21/511)	18 (92/511)	
T3 (%)	41 (207/511)	40 (202/511)	7 (37/511)	41 (207/511)	
T4 (%)	41 (212 /511)	38 (196/511)	9 (46/511)	41 (212/511)	
cN stage					0.86 (0.82, 0.91)
N0 (%)	37 (187/511)	40 (204/511)	7 (33/511)	38 (195/511)	
N1-3 (%)	63 (324/511)	60 (307/511)	7 (33/511)	62 (316/511)	

NOTE. — For observer 1 and observer 2, data are reported as percentages with number/number in parentheses. For Kappa value, data in parentheses are 95% CI. cT stage = T stage of CT; cN stage = N stage of CT.

Supplemental materials undergo peer review but are not copyedited, and may include typographical errors.

Table S4 Multivariable Logistic Regression Analysis for the Models to Predict MSI/dMMR EGJA

Models	β	S.E.	Wald	*OR*	*P* value
Clinical					
Sex (Female)	1.21	0.42	8.06	3.34 (1.45, 7.67)	.005
cN stage (N0)	1.61	0.42	14.37	5.01 (2.18, 11.52)	<.001
Constant	-2.41	0.38	40.85	-	<.001
Spectral CT					
CT_{40keV_VP}	-0.02	0.01	7.86	0.98 (0.97, 0.99)	.005
NID_{VP}	-9.32	2.07	20.35	0.00 (0.00, 0.01)	<.001
Constant	8.96	2.19	16.80	-	<.001
Nomogram					
Sex (Female)	1.07	0.48	5.01	2.93 (1.14, 7.50)	.03
cN stage (N0)	1.81	0.51	12.78	6.13 (2.27, 16.55)	<.001
CT_{40keV_VP}	-0.02	0.01	6.40	0.98 (0.97, 1.00)	.01
NID_{VP}	-10.33	2.44	17.94	0.00 (0.00, 0.00)	<.001
Constant	8.17	2.37	11.87	-	<.001

NOTE. — Data in parentheses are 95% CIs. cN stage = clinical N stage; VP = venous phase; NID = normalized iodine density; β = regression coefficient; S.E. = standard error of regression coefficient; OR = odds ratio.

Supplemental materials undergo peer review but are not copyedited, and may include typographical errors.

Table S5 Univariable and Multivariable Logistic Regression Analyses of Spectral CT Parameters in the Training Cohort

Variables	Univariable analysis		Multivariable analysis	
	OR	*P* value	*OR*	*P* value
Maximum diameter	1.11 (0.93, 1.32)	.24		
Arterial phase				
CT_{40keV}	0.99 (0.98, 1.00)	.01	0.99 (0.97, 1.01)	.19
CT_{50keV}	0.99 (0.97, 1.00)	.04	1.01 (0.98, 1.05)	.43
CT_{60keV}	0.99 (0.97, 1.01)	.51		
CT_{70keV}	1.00 (0.97, 1.03)	.93		
CT_{80keV}	0.99 (0.95, 1.03)	.53		
CT_{90keV}	0.99 (0.95, 1.04)	.79		
CT_{100keV}	0.97 (0.93, 1.02)	.29		
ID	0.43 (0.22, 0.83)	.01	1.13 (0.28, 4.54)	.86
NID	0.00 (0.00, 0.08)	.005	0.00 (0.00, 27.94)	.16
Z_{eff}	0.63 (0.22, 1.83)	.40		
NZ_{eff}	0.06 (0.00, 9.53)	.27		
Venous phase				
CT_{40keV}	0.99 (0.97, 1.00)	.006	0.99 (0.97, 1.00)	.03*
CT_{50keV}	0.99 (0.98, 1.01)	.21		
CT_{60keV}	0.99 (0.98, 1.01)	.48		
CT_{70keV}	1.00 (0.98, 1.02)	.68		
CT_{80keV}	1.00 (0.97, 1.02)	.97		
CT_{90keV}	1.00 (0.97, 1.03)	>.99		
CT_{100keV}	1.01 (0.98, 1.05)	.51		
ID	0.79 (0.45, 1.38)	.41		
NID	0.00 (0.00, 0.01)	<.001	0.00 (0.00, 0.01)	<.001*
Z_{eff}	0.66 (0.28, 1.59)	.36		
NZ_{eff}	0.06 (0.00, 9.53)	.27		

NOTE. — Data in parentheses are 95% CIs. Odds ratios (ORs) were derived from univariable and multivariable logistic regression analyses. Maximum diameter was the longest tumor diameter measured on the axial plane in the 40 keV VMI during the VP. OR = odds ratio; ID = iodine density; NID = normalized iodine density; Z_{eff} = effective atomic number; NZ_{eff} = normalized effective atomic number.

* Variables ultimately selected through multivariable logistic regression analysis ($P < .05$).

Supplemental materials undergo peer review but are not copyedited, and may include typographical errors.

Table S6 Comparison of Quantitative Spectral CT Parameters between MSI/dMMR and MSS/pMMR Statuses

Models	*P* value for CT_{40keV_AP}	*P* value for NID_{AP}	*P* value for CT_{40keV_VP}	*P* value for NID_{VP}
Training Cohort	.02	.003	.002	<.001
Validation cohort	.01	.04	.01	<.001
External test cohort	.03	.04	.048	.001
Prospective test cohort	.02	.047	<.001	<.001
Neoadjuvant chemotherapy cohort	.002	.046	.002	<.001

NOTE. —The Mann-Whitney *U* test was used for comparisons between the MSI/dMMR and MSS/pMMR statuses due to the nonnormal distributions of the data. AP = arterial phase; VP = venous phase; NID = Normalized iodine density.

Supplemental materials undergo peer review but are not copyedited, and may include typographical errors.

Table S7 Performance Comparison of the Spectral CT Model, the Clinical Model and the Nomogram

Models	Training Cohort		Validation cohort		External test cohort		Prospective test cohort		Neoadjuvant chemotherapy cohort	
	ΔAUC	*P* value	ΔAUC	*P* value	ΔAUC	*P* value	ΔAUC	*P* value	ΔAUC	*P* value
Spectral CT model vs. Clinical model	0.05 (-0.08, 0.18)	.42	0.11 (-0.07, 0.29)	.22	0.09 (-0.15, 0.33)	.45	0.15 (-0.02, 0.33)	.08	0.09 (-0.07, 0.26)	.28
Nomogram vs. Spectral CT model	0.07 (0.00, 0.13)	.03	0.04 (-0.05, 0.13)	.39	0.10 (-0.02, 0.21)	.09	0.04 (-0.07, 0.16)	.46	0.05 (-0.03, 0.13)	.19
Nomogram vs. Clinical model	0.12 (0.04, 0.20)	.002	0.15 (0.05, 0.25)	.003	0.19 (0.05, 0.33)	.009	0.20 (0.12, 0.27)	<.001	0.14 (0.02, 0.27)	.02

NOTE. — Data in parentheses are 95% CIs. ΔAUC = difference between the area under the receiver operating characteristic curves of the compared models.

Supplemental materials undergo peer review but are not copyedited, and may include typographical errors.

Table S8 Predictive Performance of the Combination CT_{70keV}+NCT_{70keV} and Combination CT_{70keV}+NCT_{70keV}+Clinical Model for MSI/dMMR

Models	AUC	Accuracy	Sensitivity	Specificity	Positive predictive value	Negative predictive value
Combination CT_{70keV} + NCT_{70keV}						
Training cohort	0.63 (0.52, 0.75)	75 (115/154)	49 (17/35)	82 (98/119)	45 (17/38)	84 (98/116)
Validation cohort	0.75 (0.63, 0.87)	65 (42/65)	88 (15/17)	56 (27/48)	42 (15/36)	93 (27/29)
External test cohort	0.61 (0.44, 0.78)	52 (24/46)	93 (14/15)	32 (10/31)	40 (14/35)	91 (10/11)
Prospective test cohort	0.60 (0.45, 0.76)	67 (61/91)	55 (11/20)	70 (50/71)	34 (11/32)	85 (50/59)
Neoadjuvant chemotherapy cohort	0.61 (0.46, 0.76)	83 (129/155)	45 (13/29)	92 (116/126)	57 (13/23)	88 (116/132)
Combination CT_{70keV}+NCT_{70keV}+Clinical						
Training cohort	0.78 (0.69, 0.87)	81 (125/154)	60 (21/35)	87 (104/119)	58 (21/36)	88 (104/118)
Validation cohort	0.83 (0.71, 0.95)	78 (51/65)	76 (13/17)	79 (38/48)	57 (13/23)	90 (38/42)
External test cohort	0.80 (0.65, 0.94)	78 (36/46)	67 (10/15)	84 (26/31)	67 (10/15)	84 (26/31)
Prospective test cohort	0.78 (0.65, 0.90)	73 (66/91)	80 (16/20)	70 (50/71)	43 (16/37)	93 (50/54)
Neoadjuvant chemotherapy cohort	0.74 (0.63, 0.86)	65 (101/155)	79 (23/29)	62 (78/126)	32 (23/71)	93 (78/84)

NOTE. — For area under the receiver operating characteristic curve (AUC), data in parentheses are 95% CIs. For accuracy, sensitivity, specificity, positive predictive value, and negative predictive value, data are reported as percentages with number/number in parentheses. NCT_{70keV} = normalized CT_{70keV}.

Supplemental materials undergo peer review but are not copyedited, and may include typographical errors.

Table S9 Performance Comparison of Combination CT_{70keV}+NCT_{70keV}+Clinical and Nomogram Model

Training Cohort		Validation cohort		External test cohort		Prospective test cohort		Neoadjuvant chemotherapy cohort	
ΔAUC	*P* value	ΔAUC	*P* value	ΔAUC	*P* value	ΔAUC	*P* value	ΔAUC	*P* value
0.10 (0.02, 0.18)	.02	0.04 (-0.09, 0.17)	.52	0.11 (-0.00, 0.23)	.06	0.11 (0.03, 0.20)	.01	0.12 (-0.02, 0.25)	.10

NOTE. — Data in parentheses are 95% CI. ΔAUC = difference between the area under the receiver operating characteristic curves of the compared models. NCT_{70keV} = normalized CT_{70keV}.

Supplemental materials undergo peer review but are not copyedited, and may include typographical errors.

Table S10 Predictive Performance of the Biopsy and the Biopsy-nomogram Model for MSI/dMMR Status

Models	AUC	Accuracy	Sensitivity	Specificity	Positive predictive value	Negative predictive value
Biopsy						
Training cohort	0.88 (0.81, 0.95)	90 (124/138)	85 (28/33)	91 (96/105)	76 (28/37)	95 (96/101)
Validation cohort	0.86 (0.75, 0.97)	86 (49/57)	86 (12/14)	86 (37/43)	67 (12/18)	95 (37/39)
External test cohort	0.89 (0.77, 1.00)	90 (37/41)	85 (11/13)	93 (26/28)	85 (11/13)	93 (26/28)
Prospective test cohort	0.84 (0.74, 0.95)	88 (76/86)	78 (14/18)	91 (62/68)	70 (14/20)	94 (62/66)
Biopsy-nomogram						
Training cohort	0.95 (0.90, 0.99)	91 (126/138)	88 (29/33)	92 (97/105)	78 (29/37)	96 (97/101)
Validation cohort	0.94 (0.87, 1.00)	88 (50/57)	100 (14/14)	84 (36/43)	67 (14/21)	100 (36/36)
External test cohort	0.98 (0.95, 1.00)	95 (39/41)	85 (11/13)	100 (28/28)	100 (11/11)	93 (28/30)
Prospective test cohort	0.92 (0.84, 1.00)	91 (78/86)	89 (16/18)	91 (62/68)	73 (16/22)	97 (62/64)

NOTE. — For area under the receiver operating characteristic curve (AUC), data in parentheses are 95% CIs. For accuracy, sensitivity, specificity, positive predictive value, and negative predictive value, data are reported as percentages with number/number in parentheses.

Supplemental materials undergo peer review but are not copyedited, and may include typographical errors.

Table S11 Performance Comparison of the Biopsy and the Biopsy-nomogram Model

Training Cohort		Validation cohort		External test cohort		Prospective test cohort	
ΔAUC	*P* value	ΔAUC	*P* value	ΔAUC	*P* value	ΔAUC	*P* value
0.07 (0.02, 0.11)	.002	0.08 (0.01, 0.16)	.07	0.09 (0.00, 0.19)	.048	0.08 (0.00, 0.15)	.05

NOTE. — Data in parentheses are 95% CI. ΔAUC = difference between the area under the receiver operating characteristic curves of the compared models.

Supplemental materials undergo peer review but are not copyedited, and may include typographical errors.

Part III. Supplementary Figures

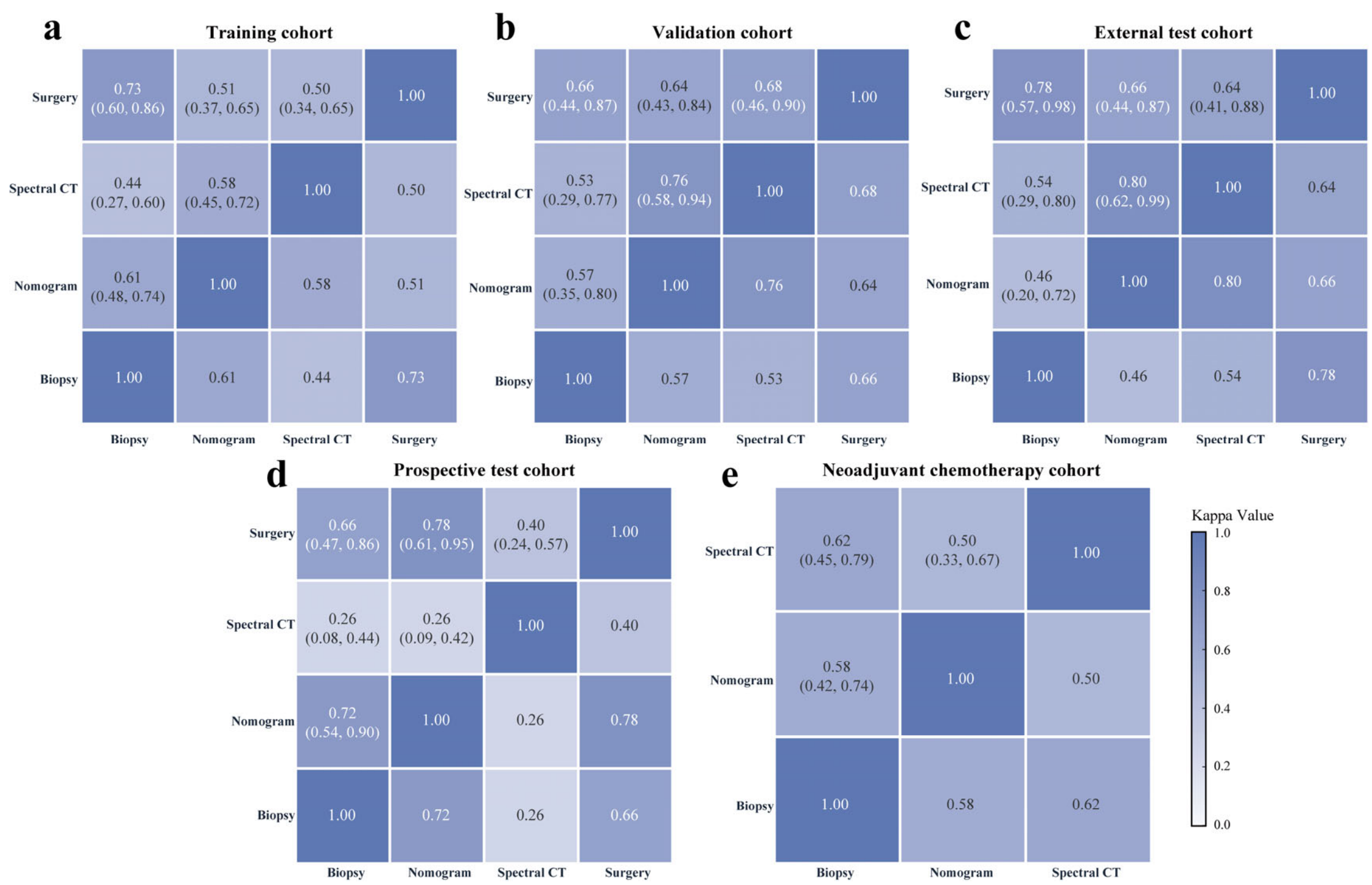

Figure S1: The diagnostic concordance in the five cohorts. The diagnostic concordance across pathological classifications of biopsy specimens, spectral CT and nomogram model predictions, and surgical resection specimens in the training cohort (a), the validation cohort (b), the external test cohort (c), the prospective test cohort (d), and the neoadjuvant chemotherapy cohort (e).

Supplemental materials undergo peer review but are not copyedited, and may include typographical errors.

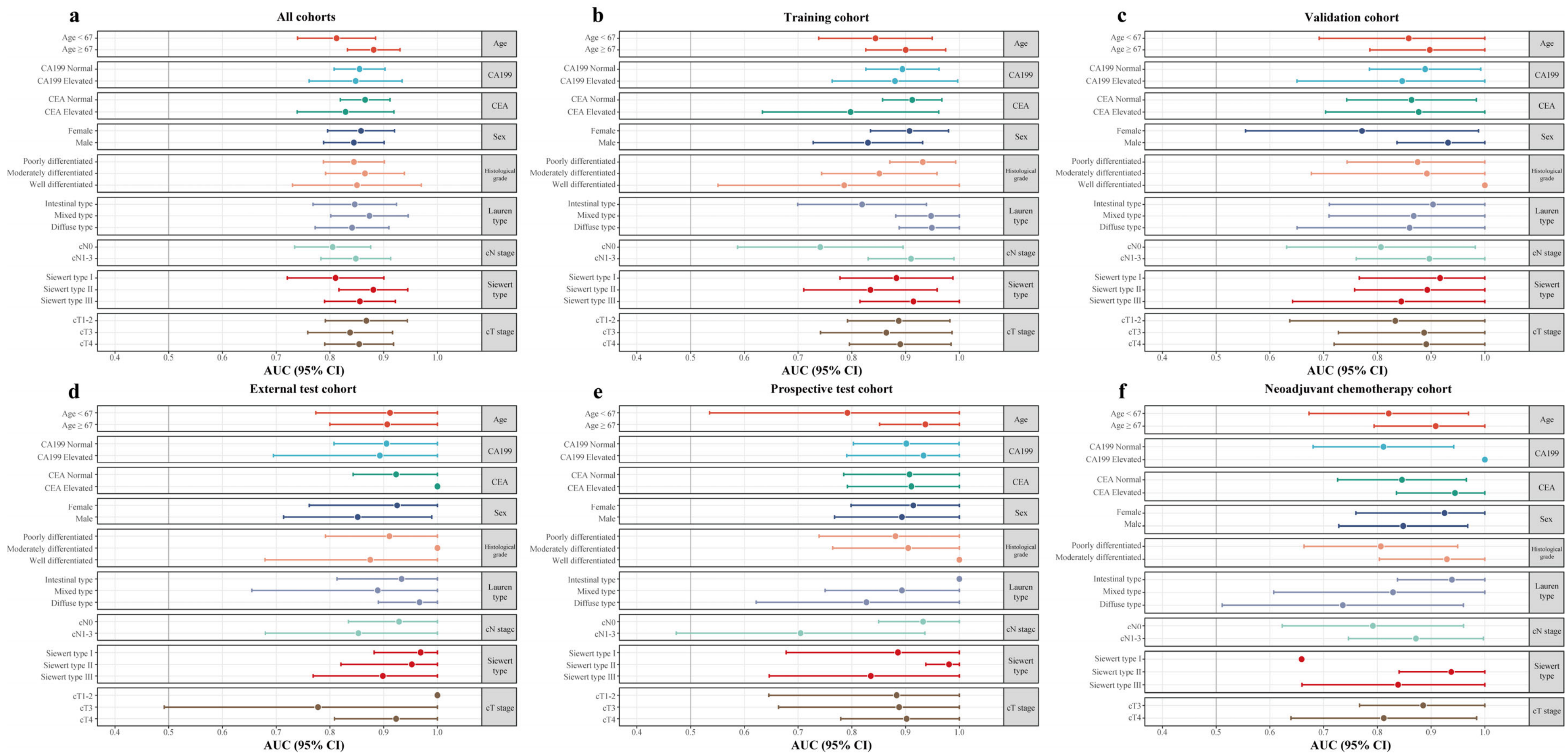

Figure S2: Subgroup analyses across the pooled cohort and all five individual cohorts. Subgroup analyses stratified by age, sex, CA199, CEA, cT stage, cN stage, Siewert type, Lauren type, and histological grade in the all cohorts (a), the training cohort (b), the validation cohort (c), the external test cohort (e), the prospective test

Supplemental materials undergo peer review but are not copyedited, and may include typographical errors.

cohort (f), and the neoadjuvant chemotherapy cohort (f). In a minority of subgroups, the AUC reached 1.00 or fell below 0.70, primarily attributable to limited sample sizes or severe category imbalances. For instance, within the validation cohort, the well-differentiated subgroup, comprised only 1 case and 8 cases respectively across the MSI/dMMR and MSS/pMMR groups. Similarly, in the external test cohort, small sample distributions were observed in the elevated CEA (3 vs.6), moderately differentiated (2 vs. 7), and cT1-2 (5 vs. 6) subgroups. This pattern persisted in the prospective cohort (well-differentiated: 3 vs. 11; intestinal type: 4 vs. 24) and the neoadjuvant chemotherapy cohort (elevated CA199: 5 vs. 27, Siewert type I: 4 vs. 38).

Supplemental materials undergo peer review but are not copyedited, and may include typographical errors.

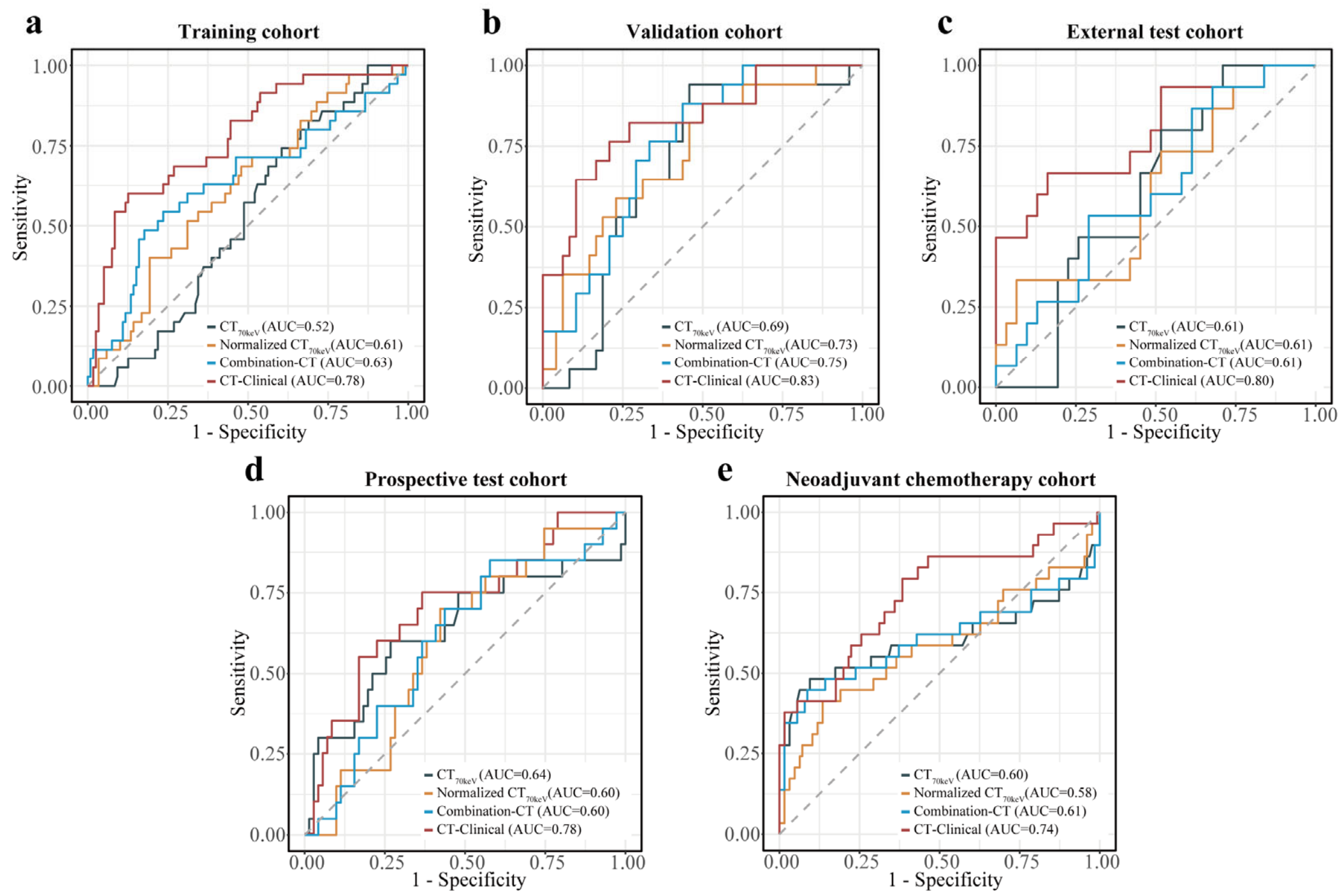

Figure S3: Receiver operating characteristic (ROC) curves of the 120kVp-like models across different cohorts. The evaluated models include the CT_{70keV} model, the normalized CT_{70keV} model, the combination-CT model (integrating CT_{70keV} and NCT_{70keV}), and the CT-Clinical model (combining CT_{70keV}, NCT_{70keV} and clinical features). ROC curves are presented for the training cohort (a), the validation cohort (b), the external test cohort (c), the prospective test cohort (d), and the neoadjuvant chemotherapy cohort (e). AUC = area under the curve.

Supplemental materials undergo peer review but are not copyedited, and may include typographical errors.

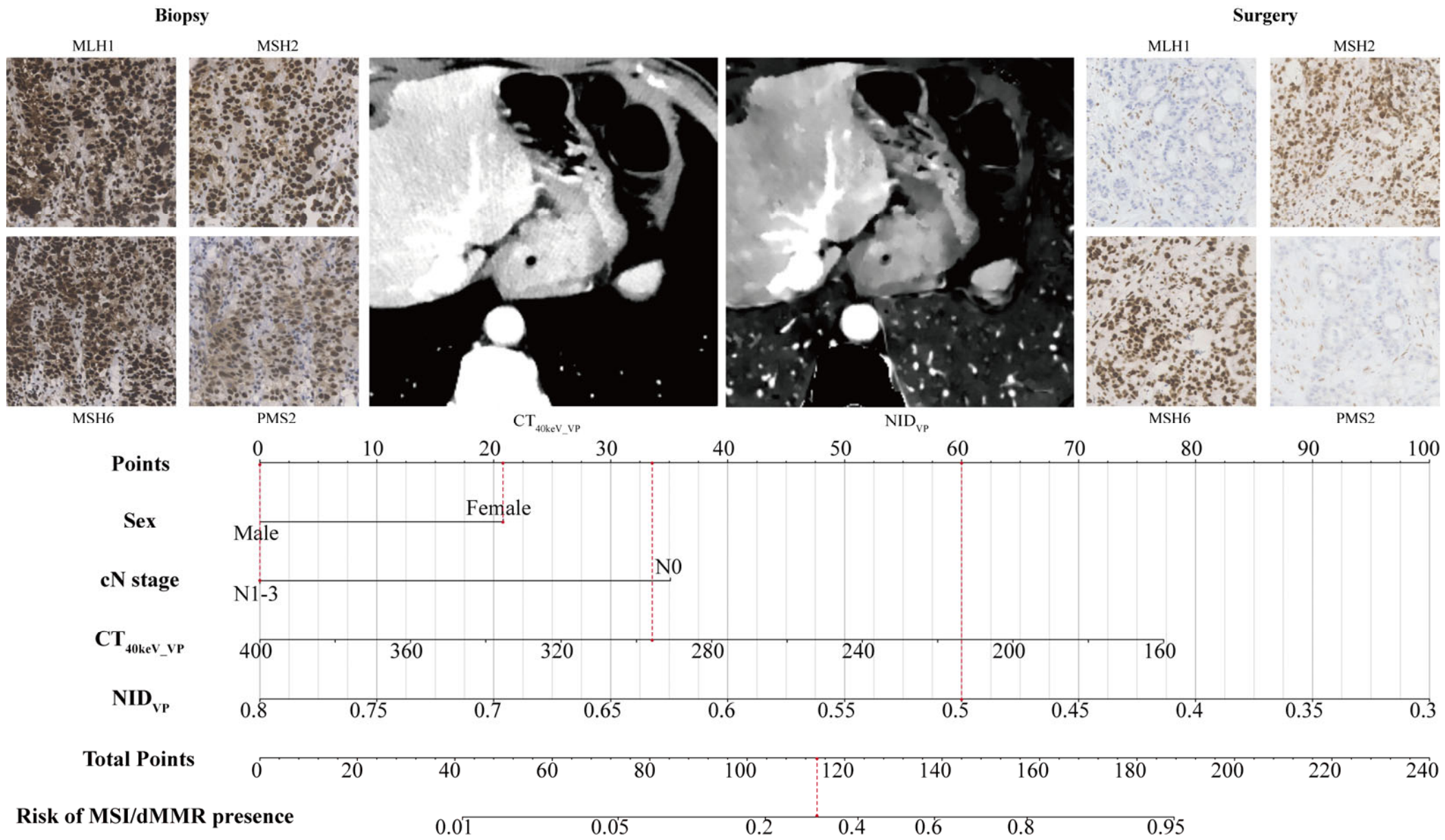

Figure S4: A retrospective patient with MSI/dMMR status correctly identified by the nomogram. A 78-year-old female patient was diagnosed with cT4N2 esophagogastric junction adenocarcinoma. Although the initial endoscopic biopsy results indicated MSS/pMMR status, the nomogram yielded 114 total points, suggesting MSI/dMMR status. The MSI/dMMR status was definitively confirmed by the surgical pathology. NID_{VP} = ID_{tumor}/ID_{aorta} in venous phase; MSI/dMMR = Microsatellite instability/ Deficient mismatch repair; MSS/pMMR = Microsatellite stable/ Proficient mismatch repair; cN stage = clinical N stage; NID = normalized iodine density; ID = iodine density; VP = venous phase; CT_{40keV_VP} = CT attenuation in 40 keV virtual monoenergetic images in venous phase; MLH1 = MutL homolog 1; MSH2 = MutS homolog 2; MSH6 = MutS homolog 6; PMS2 = post-meiotic segregation increased 2.

Supplemental materials undergo peer review but are not copyedited, and may include typographical errors.

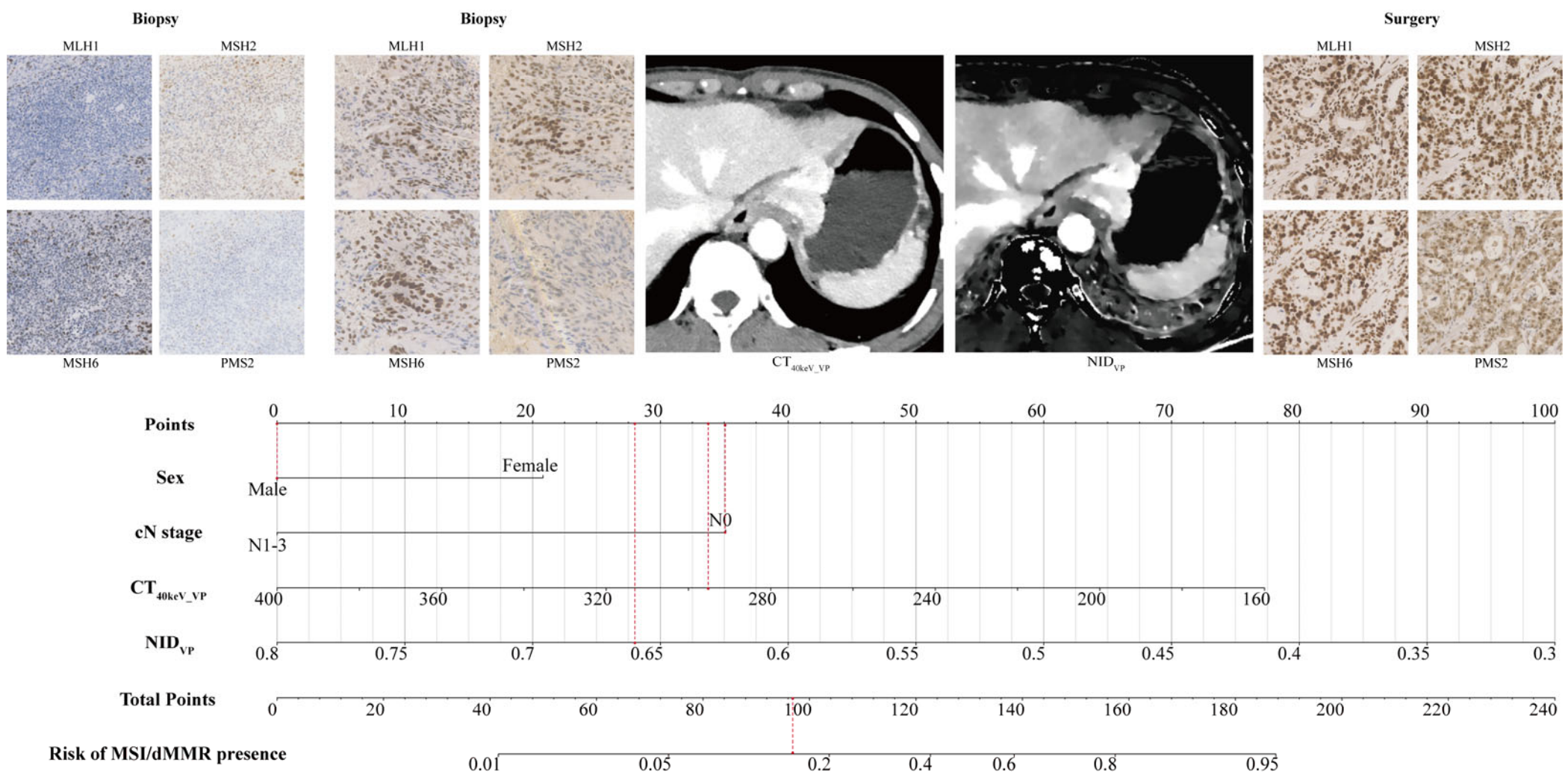

Figure S5: A prospective patient with MSS/pMMR status correctly identified by the nomogram. A 72-year-old male patient was diagnosed with cT4N0 esophagogastric junction adenocarcinoma. While the initial endoscopic biopsy suggested MSI/dMMR status, the nomogram yielded 97 total points, indicating MSS/pMMR status. Given this discordance, the clinical and pathological teams reevaluated the biopsy sections and revised the diagnosis to MSS/pMMR status. The final surgical pathology definitively confirmed MSS/pMMR status. $NID_{VP} = ID_{tumor}/ID_{aorta}$ in venous phase; MSI/dMMR = Microsatellite instability/ Deficient mismatch repair; MSS/pMMR = Microsatellite stable/ Proficient mismatch repair; cN stage = clinical N stage; NID = normalized iodine density; ID = iodine density; VP = venous phase; CT_{40keV_VP} = CT attenuation in 40 keV virtual monoenergetic images in venous phase; MLH1 = MutL homolog 1; MSH2 = MutS homolog 2; MSH6 = MutS homolog 6; PMS2 = post-meiotic segregation increased 2.

Supplemental materials undergo peer review but are not copyedited, and may include typographical errors.

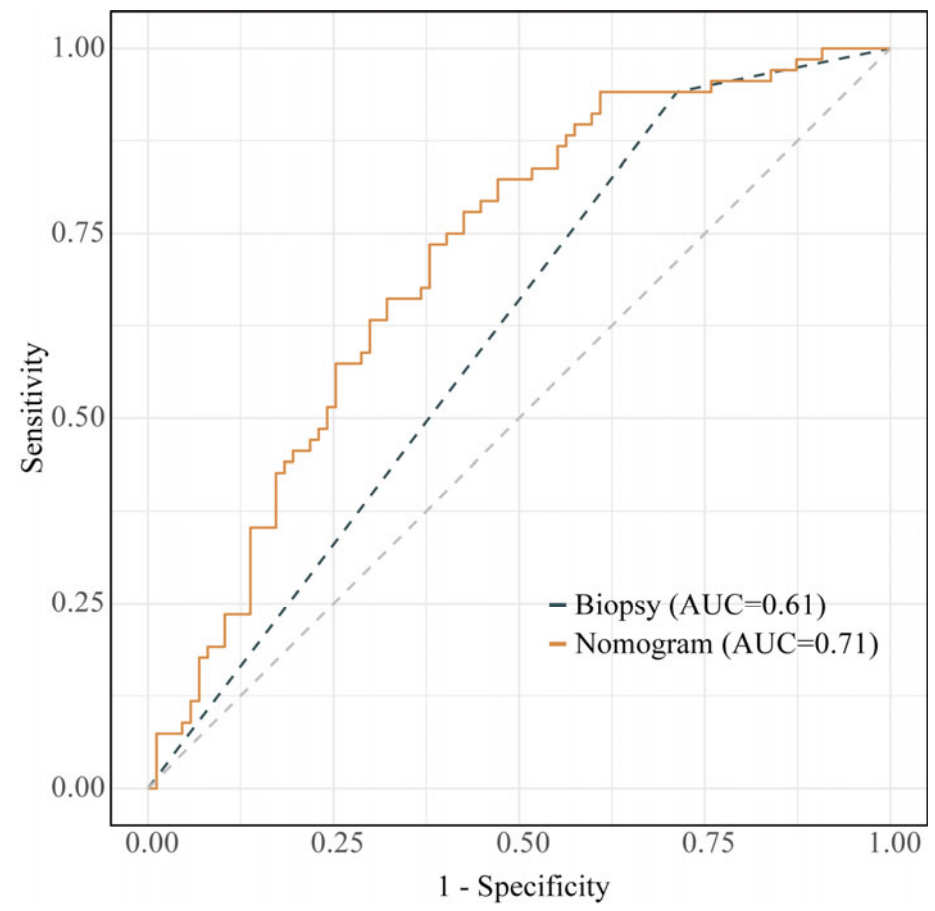

Figure S6: Receiver operating characteristic (ROC) curves of the nomogram and the biopsy result for predicting major pathological response after neoadjuvant chemotherapy. AUC = area under the curve.
